# AutoML-Driven Insights into Patient Outcomes and Emergency Care During Romania’s First Wave of COVID-19

**DOI:** 10.3390/bioengineering11121272

**Published:** 2024-12-15

**Authors:** Sonja C. S. Simon, Igor Bibi, Daniel Schaffert, Johannes Benecke, Niklas Martin, Jan Leipe, Cristian Vladescu, Victor Olsavszky

**Affiliations:** 1 Department of Dermatology, Venereology and Allergology, University Medical Center and Medical Faculty Mannheim, Heidelberg University, Theodor-Kutzer-Ufer 1-3, 68167 Mannheim, Germany; sonja.simon@umm.de (S.C.S.S.); igor.bibi@stud.uni-heidelberg.de (I.B.); daniel.schaffert@stud.uni-heidelberg.de (D.S.); kontakt@johannesbenecke.de (J.B.); niklas.martin.vhm@gmail.com (N.M.); victor.olsavszky@medma.uni-heidelberg.de (V.O.); 2 Department of Medicine V, Division of Rheumatology, University Medical Center and Medical Faculty Mannheim, Heidelberg University, Theodor-Kutzer-Ufer 1-3, 68167 Mannheim, Germany; jan.leipe@umm.de; 3 National Institute for Health Services Management, 030167 Bucharest, Romania; 4 Faculty of Medicine, University Titu Maiorescu, 031593 Bucharest, Romania

**Keywords:** automated machine learning, artificial intelligence, COVID-19, disease prediction

## Abstract

Background: The COVID-19 pandemic severely impacted healthcare systems, affecting patient outcomes and resource allocation. This study applied automated machine learning (AutoML) to analyze key health outputs, such as discharge conditions, mortality, and COVID-19 cases, with the goal of improving responses to future crises. Methods: AutoML was used to train and validate models on an ICD-10 dataset covering the first wave of COVID-19 in Romania (January–September 2020). Results: For discharge outcomes, Light Gradient Boosted models achieved an F1 score of 0.9644, while for mortality 0.7545 was reached. A Generalized Linear Model blender achieved an F1 score of 0.9884 for “acute or emergency” cases, and an average blender reached 0.923 for COVID-19 cases. Older age, specific hospitals, and oncology wards were less associated with improved recovery rates, while mortality was linked to abnormal lab results and cardiovascular/respiratory diseases. Patients admitted without referral, or patients in hospitals in the central region and the capital region of Romania were more likely to be acute cases. Finally, counties such as Argeş (South-Muntenia) and Brașov (Center) showed higher COVID-19 infection rates regardless of age. Conclusions: AutoML provided valuable insights into patient outcomes, highlighting variations in care and the need for targeted health strategies for both COVID-19 and other health challenges.

## 1. Introduction

The 2019 coronavirus disease (COVID-19) pandemic, caused by the severe acute respiratory syndrome coronavirus 2 (SARS-CoV-2), has posed unprecedented challenges to healthcare systems worldwide. The rapid spread of SARS-CoV-2 led to different governmental measures including non-medical interventions like face masks [1], social distancing and lockdowns [2]. Despite these efforts, most hospitals faced a surge in SARS-CoV-2-infected patients in the early months of the pandemic. Rising numbers of patients with COVID-19 complicated the management and treatment outcomes, emphasizing the need for predictive tools to better allocate resources and anticipate patient outcomes [3,4].

In particular, Romania, a south-eastern European country, was faced with enormous challenges due to a structurally under-resourced health system, large numbers of returning expats, and socio-cultural and religious determinants [5,6]. The first SARS-CoV-2 infection was reported in Romania on the 26th February 2020 [7], and during the first seven months, COVID-19 spread to up to 127,572 confirmed cases by 30 September 2020 [8]. Government measures in Romania in response to the accumulation of infections included restrictions on freedom of movement, closure of non-essential businesses, or isolation at home for 14 days upon arrival in Romania, to name a few [5]. Moreover, the growing number of hospitalized patients with COVID-19, along with those under suspicion of SARS-CoV-2 infection, contributed to the emergence of COVID-19 outbreaks among healthcare workers and a shortage of protective equipment and medical resources [5]. In mid-March 2020, a state of emergency in Romania was declared [9], and shortly afterwards the first COVID-19-related deaths were reported in the country [10]. Following a nationwide lockdown and the passing of a law on the wearing of surgical masks in public, the first significant relaxation measures did not begin until September 2020 [11]. After a total of five such waves of COVID-19, the state of alert was finally lifted in March 2022 [12]. Overall, the COVID-19 pandemic in Romania resulted in 65,714 deaths out of a total of 2,912,705 reported cases [8]. These worrying figures call for diversified epidemiological approaches to better understand the influences and magnitudes of such pandemic waves.

The unprecedented strain on Romania’s healthcare system during the pandemic, combined with the complexity of managing the public health response, highlights the need for advanced technological solutions to optimize care and decision-making processes. One such solution is the integration of machine learning (ML), which has demonstrated significant potential to improve patient safety [13] and increase healthcare efficiency [14]. Despite its capability to enhance clinical decision-making, ML is seldomly integrated in clinical settings, often due to lack of expertise [15]. Therefore, automated machine learning (AutoML) has emerged as a powerful approach, enabling the development and deployment of predictive models without the need for extensive manual tuning and expert knowledge [15]. AutoML incorporates a set of tools and techniques that automate the process of building, training and deploying ML models. Traditional machine learning workflows often require extensive expertise in tasks such as data pre-processing, feature engineering, model selection and hyper-parameter optimization. AutoML simplifies and automates these steps, enabling non-expert users to develop and deploy high-quality models with minimal manual intervention. For example, AutoML systems can automatically explore different model architectures, optimize hyperparameters and select the best-performing model for a given dataset, speeding up the entire modelling process. This automation is particularly beneficial in complex domains such as healthcare, where there is a need to quickly derive insights from large datasets. In the context of the COVID-19 pandemic, AutoML has been shown to generate models that can help diagnose SARS-CoV-2 infection [16,17,18,19], predict patient outcomes [16,20], forecast the spread of COVID-19 [21] and optimize hospital operations [22]. In particular, the AutoML platform DataRobot has been used in COVID-19-related research to enable automated modelling to support government decision making [23]. Specifically, DataRobot has been used to support the analysis of scientific publications [24], the planning of public health interventions [25] and the prediction of COVID-19 severity [26].

Using AutoML, we have recently uncovered relationships between patient characteristics and disease activity in psoriasis and chronic eczema, and predicted therapy changes in psoriasis and psoriatic arthritis [27,28]. In addition, automated time-series analysis allowed us to accurately predict the prevalence of the 10 most lethal diseases and parasitic diseases in Romania using the national Diagnosis Related Groups (DRG) database [29,30]. In this study, our main objectives were to analyze patient outcomes during the first wave of the COVID-19 pandemic in Romania, specifically from January 2020 to September 2020. Using DataRobot’s AutoML platform, we aimed to explore factors associated with four key outcomes: condition at discharge (cured or improved), mortality (deceased), acute or emergency cases, and confirmed COVID-19 diagnosis. These targets were chosen to provide a comprehensive understanding of patient trajectories and healthcare needs during this critical period. By focusing on these outcomes, we sought to identify patterns related to patient characteristics, healthcare resource allocation and regional variations, thereby providing insights to optimize clinical and public health responses in future pandemics or similar health crises.

To our knowledge, this is the first study to use AutoML to analyze comprehensive hospital data in Romania during the early months of the COVID-19 pandemic. While numerous studies have examined COVID-19 patient outcomes, this study is unique in applying a comprehensive AutoML approach to a nationwide dataset covering Romania’s first COVID-19 wave. Unlike many previous analyses that rely on traditional statistical methods or focus on specific subpopulations, our approach allows for unbiased, automated exploration of large-scale clinical data, identifying nuanced patterns in patient outcomes, mortality and acute case rates across diverse demographic and geographic factors. By leveraging AutoML, this study not only provides insights into the impact of COVID-19 on the Romanian healthcare system, but also demonstrates the broader potential of automated machine learning in rapidly evolving public health crises.

## 2. Materials and Methods

### 2.1. Data Source

All patients hospitalized in Romania have been classified in a DRG database since 2003 [31,32]. DRG data are reported monthly to the National Institute for Health Services Management (Romanian: Institutul Național de Management al Serviciilor de Sănătate (INMSS)), formerly the National School of Public Health, Management and Professional Development (NSPHMPDB). All patients classified into a DRG and hospitalized in Romania from January 2020 through September 2020 were included in the primary dataset. During this period, the COVID-19 pandemic began to spread in Romania. Patients with confirmed SARS-CoV-2 infection were hospitalized based on the severity of their condition, including both day hospitalization and continuous hospitalization cases. The dataset comprised 825,698 hospitalized cases for any diagnosis, including those with confirmed SARS-CoV-2 infection. A total of 34 different features were included in the primary dataset, covering sociodemographic parameters (age, sex, occupation, education level, etc.), medical information (main diagnosis, secondary diagnoses, main procedures, DRG code, etc.), hospitalization information (admission type, admission ward, days spent in intensive care unit, status at discharge, etc.) and geographic information (NUTS2 region, administrative region, etc.). The features “Patient ID” and “Hospital ID” have been pseudonymized by the National Institute for Health Services Management. The data were retrieved on 21 October 2020.

### 2.2. Data Preparation

Prior to AutoML analysis, the data were thoroughly prepared into a secondary dataset in Python using the Polars library to enable high-performance data processing [33]. In order to make the general columns, i.e., the features, understandable, they were translated from Romanian to English and in some cases renamed to facilitate the overview and interpretation of the ML results. We excluded features deemed irrelevant to our analysis, such as “unique insurance ID”, “national health insurance type ID”, and “insurance validation ID”. Next, specific feature transformations were performed. Given that SARS-CoV-2 test positivity was coded in the primary dataset within the features “admission ID” or “secondary diagnoses”, we created three new Boolean features to differentiate between various COVID-19 statuses: “has_COVID-19”, indicating a confirmed COVID-19 diagnosis; “has_COVID-19_suspected_or_performed_test”, indicating cases where there was a suspicion of COVID-19 and a test was performed; and “has_positive_test”, specifying whether a COVID-19 test result was positive. Further Boolean features were created from the categorical features “state at discharge: cured or ameliorated”, “state at discharge: deceased”, “had intensive care”, “has ventilation”, and “is Romanian”. Additional new features were created to enrich the dataset with information on the weekday of admission or discharge and information on various government responses to the COVID-19 pandemic (Table S1). Since the One Hot Encoding of secondary diagnoses created too many columns, we merged the ICD-10 codes with the systematic chapters from the ICD-10-GM document from the German Federal Institute for Drugs and Medical Devices [34] and used One Hot Encoding for these chapters. A list with all 71 features included in the final secondary dataset is found in Table S2.

### 2.3. Exploratory Data Analysis

For the identification of associations between features, we used the DataRobot’s AutoML platform. A comprehensive description of the AutoML platform’s functionality has been previously described [27,28]. In short, an initial exploratory data analysis (EDA) by the AutoML platform produces a summary of the dataset, including data quality metrics and descriptive statistics. The number of rows and columns, missing values, data type, and distribution for the top 50 items of each feature are provided. Furthermore, numerical statistics such as mean, standard deviation, median, minimum and maximum are reported for numerical features. Automatic feature transformations categorize the raw features in different types, such as numeric, categorical, Boolean, and the like. Following the results of the initial EDA, we manually improved the data quality by excluding data leakage detected and flagged by the AutoML platform. Incorrectly assigned feature categories were then adjusted. Next, four different targets were selected for this study, namely “state at discharge: cured or ameliorated”, “state at discharge: deceased”, “acute case or emergency”, and “has COVID-19”. After target selection, a second exploratory data analysis is performed. Other data quality issues are identified, such as outliers, errors in multi-category formats, inliers, excessive zeros, hidden missing values and target leakage. Details of how these issues have been addressed, including the approach to missing values, are provided in Table S3. Then, each feature in the dataset is ranked according to its importance for the target prediction. Features with low importance for the target are excluded, leading to a reduced feature list. AutoML analysis with a reduced feature list can increase accuracy and avoid errors. Therefore, we reduced the feature lists for each target based on importance and further eliminated target leakages individually for each target (Table S4).

### 2.4. Model Building, Validation, and Selection

Next, the AutoML platform trains and validates the best model for a given objective through a variety of combinations of data transformations and testing of a wide range of models. The models are automatically ranked on procedures such as boosting, bagging, random forests, kernel-based methods, generalized linear models, deep learning, and many others. Moreover, blender models can improve predictive performance and implement more accurate versions of the superior ranking models. Importantly, the dataset is divided into a training set containing approximately 65% of the data to identify relationships between the target and all features. The remaining data are divided into a validation set and a holdout set to test accuracy and rank the models. To address the issue of unbalanced data for all target features, a stratified sampling approach to data partitioning is used. This method preserves the distribution of the target feature across the training, validation and test sets, ensuring that each subset accurately represents the class proportions of the original data. This stratified partitioning helps the models to generalize better by exposing them to both majority and minority classes in all partitions. The AutoML platform also uses a technique called “smart downsampling” to efficiently handle big datasets. In this approach, the majority class is reduced to balance the dataset, allowing faster model building without sacrificing accuracy. A weight is then applied to mimic the original class distribution in the training process, ensuring that the effect of the resulting dataset is representative of the original data. Furthermore, a grid search approach is used during model training to optimize parameters for unbalanced data. The grid search is performed on a 70/30 train/test split within the training data, and after identifying the best parameter values, the final model is retrained on the full training dataset.

To reduce overfitting to the training dataset, cross-validation (CV) optimizes model selection by repeatedly splitting the training partition into different smaller training/validation subsets. For each target, we ultimately selected one of the most accurate models, focusing primarily on the 5-fold cross-validation results. For this purpose, the Area Under Curve (AUC) and the Logarithmic Loss (LogLoss) metric, widely used in ML applications [35,36], were considered (Table 1). For additional insights into the process of modelling, the DataRobot platform renders documentation for each model including a blueprint. The blueprint shows all steps performed during model building, such as pre-processing steps, modelling algorithms and post-processing steps (Figure S1).

The analysis and model training were conducted on the Amazon Web Services (AWS) cloud infrastructure in the European Union (Ireland region), ensuring compliance with General Data Protection Regulation (GDPR) regulations. This managed artificial intelligence (AI) cloud environment provided secure and scalable computational resources for efficient model training and testing.

### 2.5. ML Analysis with Feature Impact and Feature Effect

In the context of ML analysis, “Feature Impact” and “Feature Effect” are important concepts used to understand how different variables influence the prediction of a model and to ensure that the model’s decision-making process is transparent [28]. Feature Impact refers to the overall importance or contribution of a feature in determining the model decisions. By changing input data and observing the effect on a model’s predictive accuracy, the AutoML platform uses permutation or tree-based importance methods to quantify the impact of features [37]. The “Feature Impact” scores are normalized to the value of the most important feature. “Feature Effect” is used to further explore the relationship between a feature and a model’s prediction, and its “Partial Dependence” (PD) values show how changes in the feature’s values affect the prediction while holding all other features constant [38].

### 2.6. Software

Graphs were created using GraphPad Prism 10.2.3 (GraphPad software), and AutoML analysis was conducted using DataRobot’s Automatic Machine Learning (version V b82aeb).

## 3. Results

In our study, we aimed to identify patterns and relationships between patient demographics, health information including COVID-19 infection, hospital factors and disease outcomes during the first nine months of the COVID-19 pandemic in Romania. Therefore, we used AutoML to train multiple ML models, allowing them to learn from our historical data and finally selecting the most accurate one to perform the analyses. We chose the target features “state at discharge: cured or ameliorated” and “state at discharge: deceased” to specifically examine patient outcomes, “acute case or emergency” to learn about predisposing factors for emergencies, and finally “has COVID-19” to analyze risk factors for individuals and regions during this first pandemic wave in Romania.

### 3.1. Target 1: State at Discharge: Cured or Ameliorated

**Figure 1:**
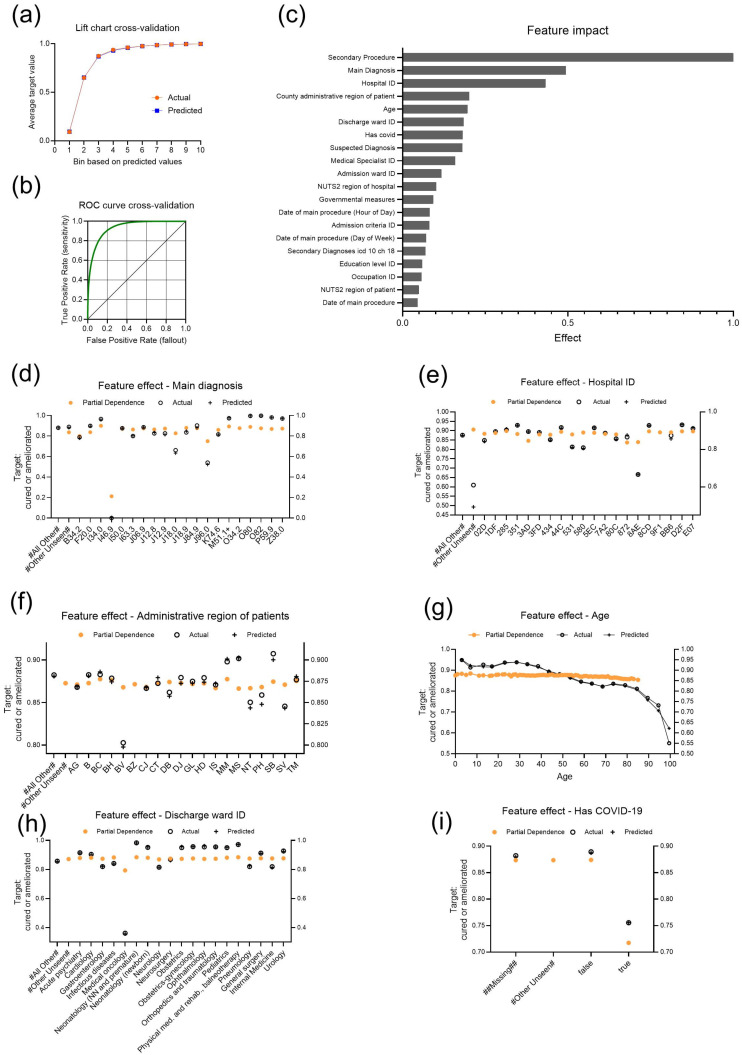
Performance indicators and insights of the model “Light Gradient Boosted Trees Classifier with Early Stopping” selected for the target “state at discharge: cured or ameliorated”. (**a**) Lift chart with actual and predicted values during cross-validation. (**b**) ROC curve (green) during cross-validation. (**c**) Feature Impact shows the top 20 features that drive model decisions, listed in descending order of normalized impact. (**d**–**i**) Plots of feature effects for (**d**) “Main diagnosis”, (**e**) “Hospital ID”, (**f**) “Administrative region of patients”, (**g**) “Age”, (**h**) “Discharge ward ID”, and (**i**) “Has COVID-19”. All feature effect plots use the y-axis to represent the target probability and the x-axis to show feature values, with partial dependence representing the marginal effect of each feature while holding others constant, and actual and predicted values included to visualize the observed and modeled outcomes.

As a first step, we analyzed the outcome of patients during the COVID-19 pandemic in Romania using cured or ameliorated (i.e., improved) discharge status as an AutoML target. A total of 577,813 cases of the CV data (Table 2) were in a cured or improved state at the time of discharge from hospital treatment. A total of 164 models were trained using binary classification (Table 1), with the final model selected, based on the strongest performance metrics, being Light Gradient Boosted Trees (LGBM) Classifier with Early Stopping. The lift chart of the selected model showed close agreement between actual and predicted values (Figure 1a). In CV, a threshold of 0.4737 resulted in an F1 score of 0.9644, a sensitivity of 0.9869 and a precision of 0.9428 (Figure 1b).

Feature impact analysis revealed that the main features associated with the target were “Secondary procedure”, “Main diagnosis”, “Hospital ID”, “County administrative region of patients”, “Age”, “Discharge ward ID” and “has COVID-19” (Figure 1c). Looking at the word cloud of the most influential feature, “secondary procedure”, the largest and most prominent procedure codes were “s08707”, which refers to “breast milk expression”, and “s06905”, which stands for “cardiopulmonary resuscitation (CPR)” (Figure S2). However, their impact on the target outcome was different. “Breast milk expression” (s08707) had a positive effect on the model’s target, suggesting a beneficial association with the outcome of interest. On the other hand, “cardiopulmonary resuscitation (CPR)” (s06905) had a negative effect on the target, indicating that patients requiring CPR had a worse prognosis or were less likely to experience recovery or improvement. Similarly, the feature effect of “Main diagnosis” showed that patients with (unspecified) cardiac arrest (I46.9) and, to a lesser extent, acute respiratory failure (J96.0), were less likely to be discharged in an improved condition (Figure 1d).

Most hospitals showed a close similarity in partial dependence, although three hospitals deviated with a lower partial dependence and therefore a lower impact on the chance of cure and improvement (Figure 1e). In addition, two hospitals had a low actual number of cured or improved patients. The individual administrative regions (counties) of Romania showed variations in partial dependence (Figure 1f). In particular, one county, Brașov (BV) in the center of Romania, had the lowest actual and predicted rate of discharges in a cured or improved state. Next, a higher age, in particular above 82 years, had a negative impact on the probability of cure and amelioration (Figure 1g). The influence of the discharge ward was particularly evident in medical oncology wards, where patients were less likely to be discharged in a cured or ameliorated state (Figure 1h). Finally, with regard to SARS-CoV-2 infection, we found that infection led to a lower probability of being discharged in a cured or improved state, with a profound difference in partial dependence compared to patients without SARS-CoV-2 infection (Figure 1i).

### 3.2. Target 2: State at Discharge: Deceased

**Figure 2:**
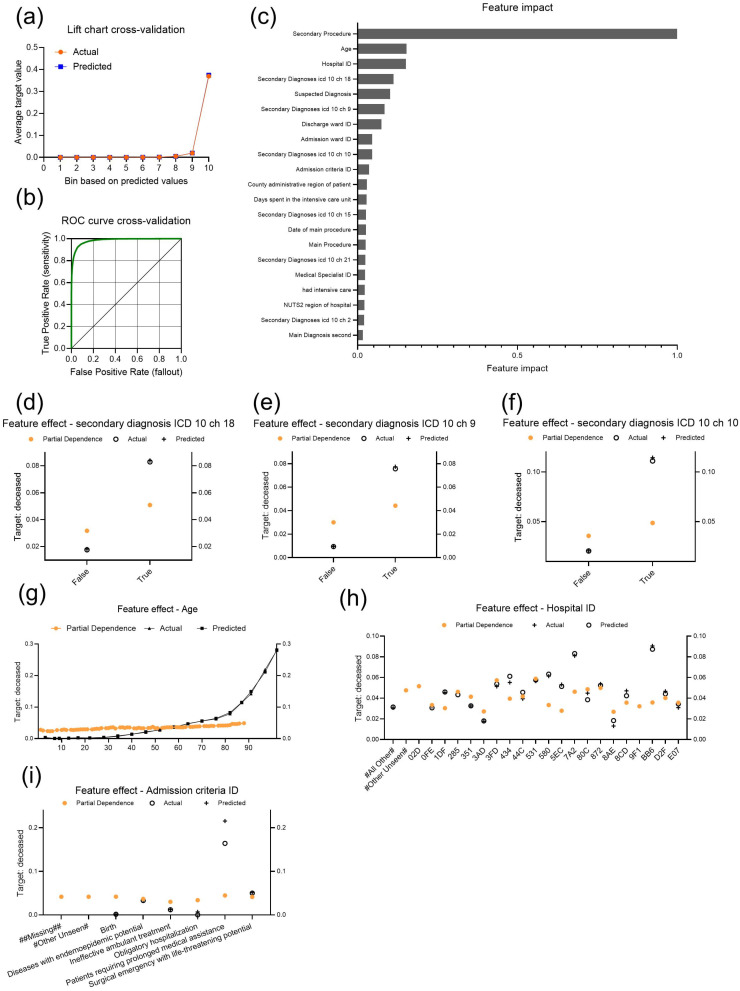
Performance indicators and insights of the model “Light Gradient Boosting on ElasticNet Predictions” selected for the target “state at discharge: deceased”. (**a**) Lift chart with actual and predicted values during cross-validation. (**b**) ROC curve (green) during cross-validation. (**c**) Feature Impact shows the top 20 features that drive model decisions, listed in descending order of normalized impact. (**d**–**i**) Plots of feature effects for (**d**) “secondary diagnosis ICD 10 chapter 18” (abnormal clinical and laboratory findings), (**e**) “secondary diagnosis ICD 10 chapter 9” (circulatory diseases), (**f**) “secondary diagnosis ICD 10 chapter 10” (respiratory diseases), (**g**) “Age”, (**h**) “Hospital ID”, and (**i**) “Admission criteria ID”. All feature effect plots use the y-axis to represent the target probability and the x-axis to show feature values, with partial dependence representing the marginal effect of each feature while holding others constant, and actual and predicted values included to visualize the observed and modeled outcomes.

Next, we chose death as a target in order to further observe influences on this specific patient outcome. A total of 22,788 patients were recorded as deceased in the CV partition (Table 2). From 175 trained models (Table 1), the chosen Light Gradient Boosting on ElasticNet Predictions demonstrated accuracy as evidenced by actual and predicted values closely aligned to each other in the lift chart (Figure 2a). A threshold of 0.4225 resulted in an F1 score of 0.7545, sensitivity of 0.6909 and precision of 0.831 in CV (Figure 2b).

The features with the greatest impact on the target were “Secondary procedure”, “Age”, “Hospital ID”, “Secondary diagnosis ICD 10 chapter 18” (symptoms, signs and abnormal clinical and laboratory findings not elsewhere classified as secondary diagnosis), “Suspected diagnosis”, “Secondary diagnosis ICD 10 chapter 9” (diseases of the circulatory system as a secondary diagnosis), or “secondary diagnosis ICD 10 chapter 10” (diseases of the respiratory system as a secondary diagnosis) and “Admission criteria” among others (Figure 2c). In this case, the procedures “advice and education on self-medication” (s04607) and “medical letter” (f00107) were frequently associated with the target outcome, but did not exert a strong influence (Figure S3). In contrast, “cardiopulmonary resuscitation” (s06905) had a strong effect on the target outcome. For patients with abnormal clinical and laboratory findings, the partial dependence and probability of death as an outcome were higher than for patients without this secondary diagnosis (Figure 2d). The same is true for patients with a secondary diagnosis of circulatory or respiratory disease, who were more likely to die (Figure 2e,f). As expected, the likelihood of dying with increasing age was evidenced by a slight increase in partial dependency and, more obviously, in actual and predicted values (Figure 2g). In this case, different hospitals showed more variation in partial dependence and even more variation in the actual number of patients who died (Figure 2h). In terms of admission criteria, patients requiring prolonged medical care and patients classified as surgical emergencies with life-threatening potential were more likely to die, as evidenced by slightly higher partial dependency (Figure 2i). However, the actual values show the greatest difference, with a high incidence of death among patients requiring prolonged medical assistance.

### 3.3. Target 3: Acute Case or Emergency

**Figure 3:**
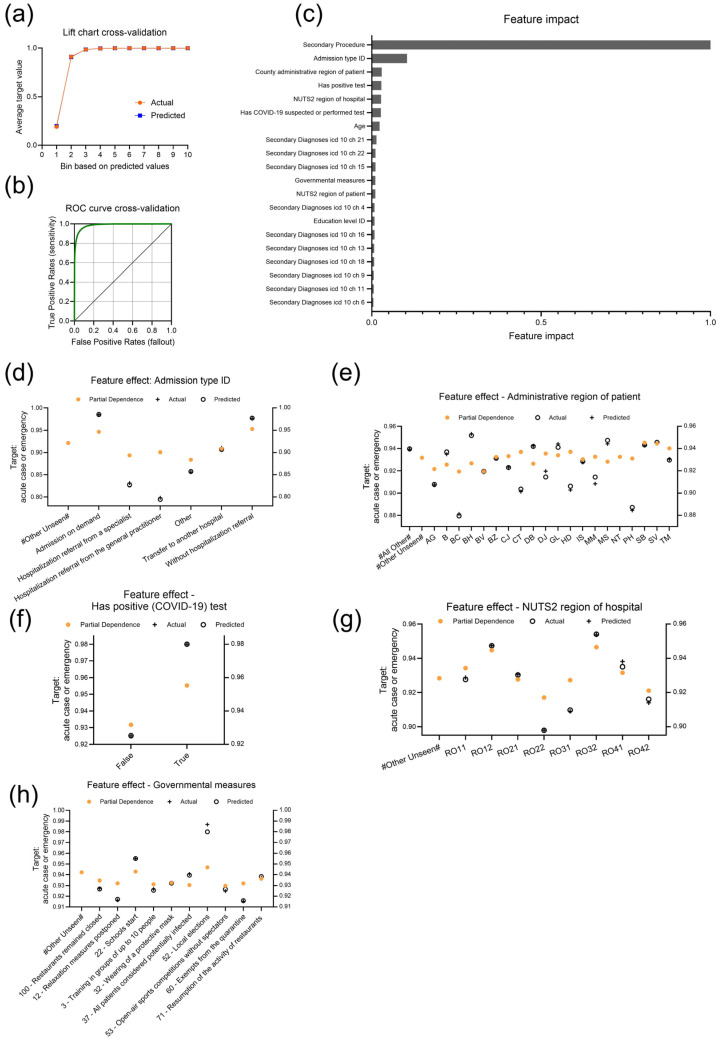
Performance indicators and insights of the model “Generalized Linear Model (GLM) (Bernoulli distribution) blender” selected for the target “acute case or emergency”. (**a**) Lift chart with actual and predicted values during cross-validation. (**b**) ROC curve (green) during cross-validation. (**c**) Feature Impact shows the top 20 features that drive model decisions, listed in descending order of normalized impact. (**d**–**h**) Plots of feature effects for (**d**) “Admission type ID” (**e**) “Administrative region of patient”, (**f**) “Has positive test” (COVID-19), (**g**) “NUTS2 region of hospital”, (**h**) “Governmental measures”. All feature effect plots use the y-axis to represent the target probability and the x-axis to show feature values, with partial dependence representing the marginal effect of each feature while holding others constant, and actual and predicted values included to visualize the observed and modeled outcomes.

We further aimed to identify patient characteristics and external factors predisposing for acute cases or emergencies. In the CV partition, a total of 615,980 cases were identified as acute or emergency (Table 2). Through AutoML analysis, 119 models were trained (Table 1), with the selected Generalized Linear Model (GLM) (Bernoulli distribution) blender showing an upward trend in the lift chart with aligned actual and predicted values (Figure 3a). The GLM blender model combined the eXtreme Gradient Boosted Trees Classifier with Early Stopping (Fast Feature Binning), the eXtreme Gradient Boosted Trees Classifier with Early Stopping (Fast Feature Binning) and Unsupervised Learning Features and the Light Gradient Boosting on ElasticNet Predictions model. The CV showed an F1 score of 0.9884, a sensitivity of 0.9934 and a precision of 0.9834 at a threshold of 0.5254 (Figure 3b).

The features with the greatest impact on whether a case is acute or emergency were “Secondary procedure” and “Admission type ID” (Figure 3c). The most prominent procedure code was “routine preoperative anesthetic evaluation” (s00201), being the most frequently occurring feature in this model and having a high influence on the target (Figure S4). Admissions were categorized as admission on demand, referral from specialist, referral from general practitioner, transfer to another hospital, admission without hospitalization referral and other. As can be seen from the partial dependence plot, admissions on demand or admissions without referral are associated with a higher likelihood of acute or emergency cases (Figure 3d), which is in line with expectations, as these types of admissions often involve patients seeking immediate or unplanned care. Other features influencing the target were “County administrative region of the patient”, “Has positive test” (COVID-19), “NUTS2 region of the hospital” and “Governmental measure” (Figure 3c).

Different counties of patients showed differences in partial dependence, with higher probabilities for acute cases (Figure 3e). For example, the counties Sibiu (SB), Suceava (SV) and Timiş (TM) showed a higher partial dependence compared to the counties Argeş (AG), Bacău (BC) or Braşov (BV). A positive test result for SARS-CoV-2, as expected, had a positive impact on the classification as emergency (Figure 3f). Interestingly, hospitals in the NUTS2 regions RO12, representing the central region, and RO32, representing the capital region, were more likely to have emergencies (Figure 3g). Finally, government actions, such as the start of school on 14 September 2020 and/or local elections on 27 September, (Table S2) affected the likelihood of hospitalization being an acute case or emergency, considering both partial dependence and actual values (Figure 3h).

### 3.4. Target 4: Has COVID-19

**Figure 4:**
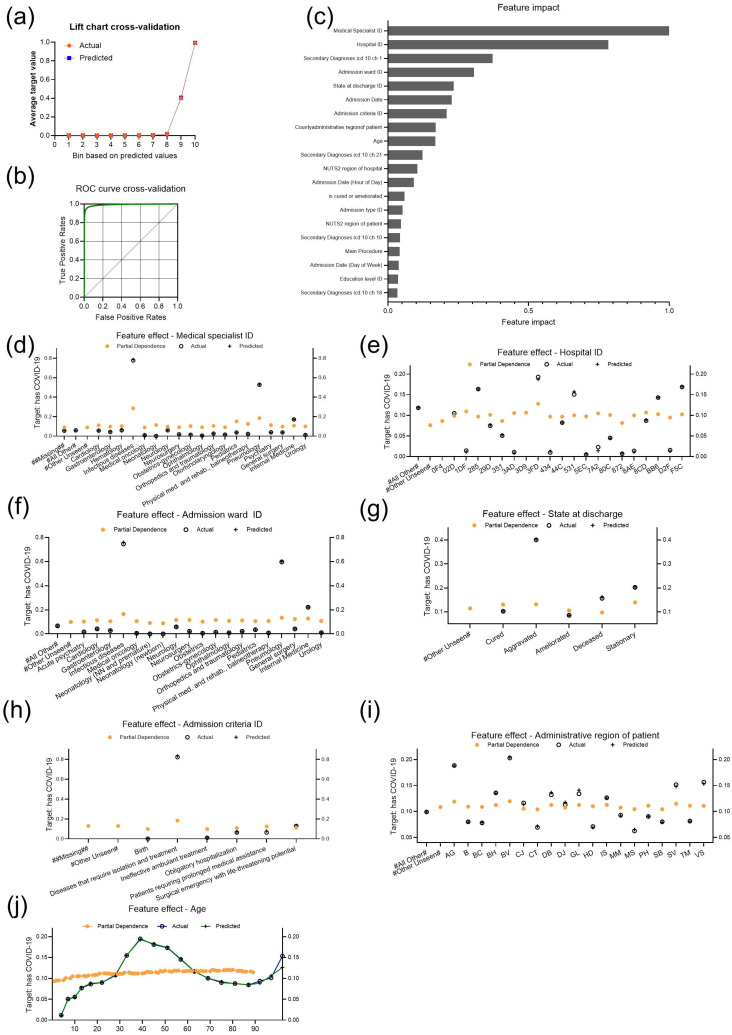
Performance indicators and insights of the model “Average (AVG) blender” selected for the target “has COVID-19. (**a**) Lift chart with actual and predicted values during cross-validation. (**b**) ROC curve (green) during cross-validation. (**c**) Feature Impact shows the top 20 features that drive model decisions, listed in descending order of normalized impact. (**d**–**j**) Plots of feature effects for (**d**) “Medical specialist ID” (**e**) “Hospital ID”, (**f**) “Admission ward ID”, (**g**) “State at discharge”, (**h**) “Admission criteria ID”, (**i**) “Administrative region of patient “, and (**j**) “Age”. All feature effect plots use the y-axis to represent the target probability and the x-axis to show feature values, with partial dependence representing the marginal effect of each feature while holding others constant, and actual and predicted values included to visualize the observed and modeled outcomes.

The final aim of the study was to investigate associations between COVID-19 itself and other relevant features of the dataset. A total of 63,460 cases were classified as SARS-CoV-2 infection in the CV partition (Table 2). The selected model from a total of 117 trained models was an average (AVG) blender combining the Light Gradient Boosted Trees Classifier with Early Stopping, the Light Gradient Boosting on ElasticNet Predictions and the eXtreme Gradient Boosted Trees Classifier with Early Stopping (Fast Feature Binning) models with closely aligned predicted and actual values in the lift chart (Figure 4a, Table 1). Setting the threshold in CV at 0.475 resulted in an F1 score of 0.923, a sensitivity of 0.9064 and a precision of 0.9402 (Figure 4b).

The first two features that had an impact on COVID-19 were “Medical specialist ID” and “Hospital ID” (Figure 4c). Other features influencing the target were “Admission ward ID”, “State at discharge”, “Admission criteria ID”, “Administrative region of patient” or “Age”. Specialists in infectious diseases and pneumology were more strongly associated with the target (Figure 4d). The partial dependence was consistent across hospitals, with only one hospital having a higher partial dependence and a much higher actual number of COVID-19 cases (Figure 4e). Similar to the medical specialties, the corresponding infectious diseases and, to a lesser extent, pneumology wards had higher partial dependence (Figure 4f). We also found that during this first wave of the pandemic in Romania, patients with SARS-CoV-2 infection were most likely to be discharged in a worsened or hospitalized condition, or to die, as evidenced by actual numbers (Figure 4g). However, the partial dependence was low for the discharge state deceased and higher for the cured. The partial dependence and the actual number were highest for patients admitted due to diseases with endemoepidemic potential that require isolation and treatment compared to other admission criteria (Figure 4h). Regarding the counties of residence of the patients, the partial dependence was higher in regions Argeş (AG) and Brașov (BV) compared to other regions, indicating a higher likelihood of SARS-CoV-2 infection, which is reflected by higher actual numbers of COVID-19 cases in AG and BV (Figure 4i). Furthermore, the partial dependence analysis of the “age” feature showed a consistent pattern (Figure 4j). However, the actual values confirmed that the highest number of SARS-CoV-2 infections occurred in people aged 30 to 60 years and the lowest number in patients under 10 years of age.

## 4. Discussion

To our knowledge, this is the first study to apply AutoML to a nationwide ICD-10 dataset covering COVID-19 disease. By analyzing retrospective data from the first wave of the COVID-19 pandemic in Romania, we identified key patterns associated with clinical outcomes, the prevalence of medical emergencies and SARS-CoV-2 infection itself. These findings provide novel insights into the broader impact of the early pandemic on public health and healthcare systems.

One of the most significant findings of the selected Light Gradient Boosted Trees Classifier with Early Stopping for the target “state at discharge: cured or ameliorated” was the association between SARS-CoV-2 infection and a reduced likelihood of being discharged in a cured or improved state. This is consistent with previous studies showing that COVID-19 patients, particularly those with co-morbidities, have longer hospital stays and poorer outcomes due to the severe respiratory and systemic effects of the virus [39]. In addition, our findings support the notion that older age is a significant predictor of adverse outcomes, particularly in patients over 82 years of age, which is consistent with global reports highlighting the increased vulnerability of older populations during the pandemic [40]. Conversely, a favorable outcome for younger SARS-CoV-2-infected patients has been reported in other countries [41]. It also emerged that individual hospitals were important determinants, with some hospitals showing lower partial dependence on the outcome of cured or improved discharge. This suggests differences in resource availability, quality of care or regional variations in the impact of COVID-19, which is consistent with similar studies highlighting the unequal burden on healthcare systems in different geographical regions [42]. Notably, in our analyzed dataset, Brașov (BV) stands out for having the lowest actual and predicted rate of discharges of patients in a cured or improved state. A key feature strongly associated with improved patient outcomes was the main diagnosis. In particular, certain conditions, such as cardiac arrest and acute respiratory failure, were associated with poorer recovery rates. This is consistent with the high morbidity and mortality commonly seen in patients with cardiac arrest, highlighting the critical and life-threatening nature of this condition and its profound impact on prognosis. In addition, while acute respiratory failure remained a very serious condition, particularly during the COVID-19 pandemic, the model indicated that the chances of recovery or improvement were slightly higher compared to those with cardiac arrest. Other factors influencing recovery included the ward of discharge. Patients in oncology wards had significantly worse outcomes due to the severity of their illness. Oncological diseases naturally carry a higher risk of poor outcomes, but a study in Romania highlighted increased mortality in cancer patients with concomitant SARS-CoV-2 infection [43]. In addition, delayed diagnosis leading to more advanced stages of disease [44] and reduced initiation of new oncological treatments [45] may have worsened outcomes.

As a consequential step, we used AutoML to focus on patient mortality and identified key findings during the early stages of the COVID-19 pandemic in Romania. In particular, excess mortality between March and December 2020 was more than double the reported COVID-19 deaths [46]. However, hospital deaths from the ten deadliest non-COVID-19 diseases decreased slightly compared to 2019 [46], suggesting that some deaths may not have been counted as COVID-related due to underreporting or indirect pandemic effects. AutoML, using the Light Gradient Boosting on ElasticNet Predictions, helped to identify factors influencing mortality and improve understanding of COVID-related deaths. Our analysis showed that both cardiopulmonary resuscitation and cardiovascular and respiratory disease, as well as abnormal clinical and laboratory findings, were significant predictors of increased mortality. This is consistent with global data showing that patients with pre-existing cardiovascular [47,48] and respiratory diseases [49] were disproportionately affected by severe COVID-19 outcomes. This is even more meaningful when considering that COVID-19 mortality is strongly associated with respiratory failure [50]. Unsurprisingly, age emerged as a critical factor, with the risk of mortality rising sharply in older patients.

Certain hospitals and admission criteria had a significant effect on survival. Patients admitted for surgical emergencies with life-threatening potential were more likely to die. While most elective surgeries were cancelled worldwide [51], including in Romania [52], studies showed longer delays in life-saving procedures such as primary percutaneous coronary intervention during the pandemic [53], suggesting a worse prognosis for emergency surgery patients. In addition, patients requiring prolonged medical care, often with chronic conditions, faced higher mortality. Disruption of chronic treatments and reduced adherence during the pandemic [54] may have increased the risk of complications and death in these patients.

In Romania, acute hospital admissions decreased by almost 40% in 2020 compared to 2019, with the sharpest decrease occurring in March during the national state of emergency [46]. Restrictions on hospital admissions led to an 80% reduction in planned hospitalizations compared to February 2020 [46]. Similar reductions in acute hospitalizations were reported in other countries during the early months of the pandemic [55,56]. Despite this, a study of an emergency department found that the most common diagnoses for admissions, excluding COVID-19-related cases, remained largely unchanged from pre-pandemic patterns [57]. In Romania, the top ten diagnoses for acute hospitalizations in 2020 were also consistent with 2018 and 2019, except for COVID-19 as a new primary diagnosis [46]. Our analysis using the GLM blender identified “Admission type ID” as a key factor, showing that admissions “on demand” or “without referral” were more strongly associated with acute or emergency cases. This is consistent with the understanding that unplanned hospital visits are often emergencies due to the urgent nature of care. Changes in referral behavior during the COVID-19 pandemic may have contributed to this pattern. A Canadian study found that general practitioners (GPs) were more likely to postpone or cancel specialist appointments [58], and an English cohort study reported a 10% decrease in urgent GP referrals for cancer patients [59]. However, when patients did attend primary care, urgent referrals were similar to or higher than pre-pandemic levels [59]. Reduced access to specialists and cancelled appointments [58] probably led more patients to bypass traditional referrals, resulting in increased emergency admissions “on demand” or “without referral”.

The feature “Has positive test” significantly influenced the likelihood of patients being classified as acute or emergency. A UK study showed that 17% of hospitalized COVID-19 patients required intensive care and 55% required high-flow oxygen, highlighting the acute nature of many cases [60]. Interestingly, geographical differences in healthcare infrastructure also affected acute care needs, with COVID-19 positivity increasing emergency classification. The NUTS2 regions RO12, representing the central region, and RO32 and RO32, representing the capital region, are highlighted as having higher probabilities of acute cases and emergencies, which is consistent with published findings that these regions had higher infection rates [42]. Similarly, counties such as Timiș (TM), which is located in a more developed region, had higher COVID-19 infection rates but lower mortality due to better health infrastructure [42]. This is in accordance with our observation that Timiș had a higher partial dependence for acute cases. Of particular relevance is the finding that government actions, such as the start of school on 14 September 2020 and the local elections on 27 September, affected the likelihood of hospitalizations being acute cases or emergencies. These events likely contributed to an increase in social interactions and population mobility, which may have accelerated the spread of COVID-19, resulting in more severe cases requiring urgent medical attention. The model’s ability to capture these effects through both partial dependence and actual values highlights the interplay between public policy decisions and health outcomes during the pandemic. It should be noted, however, that these government policies coincided with the beginning of autumn, when respiratory illnesses are usually prevalent.

The final model, aimed at predicting COVID-19 cases, provided important insights into the associations between SARS-CoV-2 infection and several key features. We selected an AVG blender model, which showed excellent predictive performance with a high F1 score, precision and sensitivity. The most influential factors associated with COVID-19 included individual hospitals and the medical specialty of the admitting physician, particularly for infectious diseases and pneumology. This is consistent with the high patient loads experienced by these departments during the first wave of the pandemic [61]. Similarly, patients admitted to wards specializing in infectious diseases and pneumology were more likely to have SARS-CoV-2 infections. These findings underscore the critical role of specialists in these fields, as well as hospitals equipped to treat epidemic diseases [62]. Healthcare infrastructure is thus a key determinant of both the spread and treatment of COVID-19, with certain hospitals with these specific units being better prepared to deal with the outbreak. In addition, SARS-CoV-2-infected patients were less likely to be discharged in an improved condition, highlighting the lack of therapeutic knowledge and options at the beginning of the COVID-19 pandemic [63]. Age was also a significant factor: adults between 30 and 60 years had the highest infection rates, while children under 10 years were less affected, consistent with other epidemiological studies [64]. Counties like Argeș and Brașov have been identified as COVID-19 hotspots, indicating regional disparities in infection rates and highlighting the need for public health interventions tailored to local healthcare capacity and infection rates. On the other hand, data published in March 2020 and August 2021 identified counties such as Cluj (CJ), Timis (TM), Constanta (CT), Ilfov (IF) and Bucharest (B) as having the highest ratio of COVID-19 cases per capita, attributed to higher levels of economic activity and population interactions [42]. Overall, both sources reflect higher COVID-19 infection rates in more densely populated regions.

In comparison with similar studies from other countries, our analysis provides unique insights into the impact of COVID-19 on an under-resourced healthcare system and highlights specific regional disparities. For example, a South Korean study emphasized the role of infection sources and regional factors in patient outcomes [65], while our findings additionally underscore the compounded effect of limited healthcare infrastructure, as seen in the lower recovery rates in specific hospitals and regions such as Brașov (BV). A Tunisian study identified age and comorbidities as critical factors in COVID-19 outcomes [66], similar to our results, but our Romanian dataset further reveals a pronounced need for targeted support for older patients and those with pre-existing conditions, particularly in rural or under-resourced areas. Likewise, an Indian study linked comorbidities such as diabetes and hypertension to severe outcomes [67], aligning with our findings that highlight the heightened risk for patients with chronic conditions. Compared to other analyses, our study emphasizes the additional burden posed by healthcare limitations and regional variations in Romania, offering valuable lessons on the need for tailored interventions in under-resourced health systems during pandemic responses.

Our AutoML analysis yields several actionable insights for optimizing healthcare responses during health crises. First, prioritizing resources for high-risk groups, particularly older patients and those with cardiovascular or respiratory conditions, is essential given their elevated risk of poor outcomes. Additionally, the observed variation in recovery rates across hospitals, notably in oncology wards, underscores the need for targeted support and standardized care protocols in these settings. Strengthening emergency preparedness for unplanned admissions, which are frequently associated with acute cases, could improve outcomes by streamlining triage and response processes. Our findings also suggest the importance of tailored public health interventions in regions with elevated COVID-19 prevalence and acute cases. Enhanced infection control measures and preventive strategies in hospitals, especially in high-positivity areas, could alleviate strain on emergency services. Furthermore, proactive planning around large-scale events, which were associated with increased acute hospitalizations, may help mitigate surges in healthcare demand. Collectively, these strategies, rooted in specific data patterns from our analysis, offer targeted approaches for improving patient care and resource allocation during pandemics and similar public health challenges.

ML in the context of COVID-19 often focuses on epidemic detection and spread prediction using conventional ML workflows, such as decision trees, support vector machines (SVMs) and neural networks [68,69]. While these models are valuable for forecasting, they often require extensive manual pre-processing and domain expertise, which AutoML effectively bypasses in our study. Therefore, the automation of feature engineering and model optimization provides a scalable solution for such analyses. To provide an objective comparison of our AutoML-derived models with other machine learning approaches in COVID-19 research, we took into consideration key performance metrics such as the F1 score and AUC. Our LGB classifier achieved an F1 score of 0.9644 for targeting cured or ameliorated discharge outcomes and 0.7545 for mortality, while an AVG blender combining multiple boosting and elastic net models achieved an F1 score of 0.923 for COVID-19 diagnosis. Our results demonstrate high accuracy across diverse clinical targets. In comparison, Raman et al. used random forests and gradient boosting models to predict disease severity at hospital admission, reporting F1 scores between 0.81 and 0.85 [70]. Similarly, Cho et al. developed an ML model for early detection of COVID-19 outbreaks, achieving an F1-score of 0.95 [68]. In addition, Burdick et al. used ML techniques to predict respiratory decompensation in COVID-19 patients, achieving an area under the receiver operating characteristic curve (AUC) of 0.78 [71]. Finally, Ramírez-del Real et al. developed models to identify individual factors associated with COVID-19 infection, reporting an AUC of 0.85 [69]. By contrast, our study simultaneously addresses multiple clinically relevant targets—recovery, mortality, acute case identification and likelihood of COVID-19 infection—achieving AUC values of up to 0.9854, for example, with our GLM blender for acute cases. While differences in datasets and target definitions must be taken into account, the higher or comparable performance of our models across multiple targets demonstrates the robustness of the AutoML approach.

A major limitation of this study is its reliance on historical data from a single pandemic wave. The dynamics of COVID-19 have evolved over time, and factors influencing outcomes may be different in later waves. The study also does not fully account for variations in hospital resource allocation or care protocols, which may have affected the results. Finally, although the AutoML approach provided robust predictions, model performance may vary between healthcare settings with different infrastructures. Despite these limitations, this study successfully identified key factors influencing patient outcomes and COVID-19 cases during the first wave of the COVID-19 pandemic in Romania, using AutoML. These findings may inform future healthcare strategies and resource allocation during public health crises. However, further research including data from later waves and different healthcare settings is needed to generalize the results.

## 5. Conclusions

This study highlights the potential of AutoML to provide actionable insights during public health crises. By analyzing Romania’s first COVID-19 wave using a comprehensive ICD-10 dataset, we identified key factors influencing patient outcomes, mortality and acute cases. Older age, oncology units and certain hospitals were associated with poorer recovery, while mortality correlated with abnormal laboratory results and pre-existing conditions. Regional disparities and the impact of government policies on acute admissions further highlight the need for tailored health strategies.

These novel findings demonstrate the value of AutoML in improving decision making and optimizing resource allocation in healthcare, particularly in under-resourced systems. Future research should extend this approach to other settings and outbreaks to validate and broaden its applicability.

## Figures and Tables

**Table 1:** Selected model performance metric scores shown for cross-validation partition. Displayed metrics include weighted AUC (Area Under the (ROC) Curve), weighted LogLoss (Logarithmic Loss), weighted RMSE (Root Mean Squared Error), weighted FVE Binomial (Fraction of Variance Explained) and weighted Max MCC (Maximum Matthews correlation coefficient). Weights are the result of smart downsampling, a dataset reduction technique.

Target	Compared Models	Selected Model	Weighted AUC	Weighted LogLoss	Weighted RMSE	Weighted FVE Binomial	Weighted Max MCC
Target 1: state at discharge: cured or ameliorated	164	Light Gradient Boosted Trees Classifier with Early Stopping	0.9333	0.1785	0.2236	0.5238	0.6787
Target 2: state at discharge: deceased	175	Light Gradient Boosting on ElasticNet Predictions	0.9836	0.0453	0.1097	0.6986	0.7513
Target 3: acute case or emergency	119	Generalized Linear Model (Bernoulli Distribution) blender (eXtreme Gradient Boosted Trees Classifier with Early Stopping (Fast Feature Binning), eXtreme Gradient Boosted Trees Classifier with Early Stopping (Fast Feature Binning) and Unsupervised Learning Features, Light Gradient Boosting on ElasticNet Predictions)	0.9854	0.0633	0.1306	0.7512	0.8264
Target 4: has COVID-19	117	AVG blender (Light Gradient Boosted Trees Classifier with Early Stopping, Light Gradient Boosting on ElasticNet Predictions, eXtreme Gradient Boosted Trees Classifier with Early Stopping (Fast Feature Binning))	0.9919	0.0510	0.1130	0.8485	0.9147

**Table 2:** The distribution of the target feature categories in the whole dataset was calculated during the data preparation phase, while the distributions for the 5-fold cross-validation and hold-out partitions were derived using the weighted coefficients applied during the smart downsampling process. By default, the AutoML platform uses a fivefold cross-validation scheme along with a separate 20% hold-out partition. The categories of each target feature are listed alongside their corresponding counts (n) and proportions (%).

Target	n; % (Whole Secondary Dataset)	n; % (All 5-Folds of Cross-Validation)	n; % (Holdout)
Target 1: state at discharge:			
cured or ameliorated	723,423; 87.61%	577,813; 87.60%	144,453; 87.60%
not cured or ameliorated	102,275; 12.39%	81,820; 12.40%	20,455; 12.40%
Target 2: state at discharge:			
deceased	28,485; 3.45%	22,788; 3.45%	5697; 3.45%
not deceased	797,213; 96.55%	636,687; 96.55%	159,171; 96.55%
Target 3:			
acute case or emergency	767,518; 92.95%	615,980; 92.98%	153,994; 92.98%
not an acute case or emergency	58,180; 7.05%	46,544; 7.02%	11,636; 7.02%
Target 4:			
has COVID-19	79,325; 10.52%	63,460; 10.53%	15,865; 10.53%
no COVID-19 infection	674,937; 89.48%	539,086; 89.47%	134,772; 89.47%

## Data Availability

The primary dataset used in this study is owned by the National Institute for Health Services Management, a Romanian institution responsible for health-related research and publications. Due to institutional policy and data protection regulations, the primary dataset is not publicly available. However, a processed secondary dataset derived from the primary data for the purposes of this study may be available upon reasonable request. Interested parties should contact the National Institute for Health Services Management, providing a clear justification for their request, including the intended use of the data. Access to the secondary dataset will be granted at the discretion of the Institute and in accordance with relevant data sharing and ethical guidelines.

## Supplementary Materials

The following supporting information can be downloaded at: https://www.mdpi.com/article/10.3390/bioengineering11121272/s1, Figure S1: Model blueprints for (a) the selected model “Light Gradient Boosted Trees Classifier with Early Stopping” for “Target 1: state at discharge: cured or ameliorated”, (b) the selected model “Light Gradient Boosting on ElasticNet Predictions” for “Target 2: state at discharge: deceased”, (c) the selected “Generalized Linear Model (Bernoulli Distribution) blender (eXtreme Gradient Boosted Trees Classifier with Early Stopping (Fast Feature Binning), eXtreme Gradient Boosted Trees Classifier with Early Stopping (Fast Feature Binning) and Unsupervised Learning Features, Light Gradient Boosting on ElasticNet Predictions) “ for “Target 3: acute case or emergency”, and (d) the selected model “AVG blender (Light Gradient Boosted Trees Classifier with Early Stopping, Light Gradient Boosting on ElasticNet Predictions, eXtreme Gradient Boosted Trees Classifier with Early Stopping (Fast Feature Binning))” for “Target 4: has COVID-19”; Figure S2: The word cloud displays the most impactful codes within the “secondary procedure” feature in relation to the target features (state at discharge: cured or ameliorated). The size of each code represents its relative influence on the model’s outcome. The color scale indicates the direction of association with the target variable: red signifies a positive association, while blue indicates a negative association. Figure S3: The word cloud displays the most impactful codes within the “secondary procedure” feature in relation to the target features (state at discharge: deceased). The size of each code represents its relative influence on the model’s outcome. The color scale indicates the direction of association with the target variable: red signifies a positive association, while blue indicates a negative association. Figure S4: The word cloud displays the most impactful codes within the “secondary procedure” feature in relation to the target features (acute case or emergency). The size of each code represents its relative influence on the model’s outcome. The color scale indicates the direction of association with the target variable: red signifies a positive association, while blue indicates a negative association. Table S1: COVID-19 Pandemic Governmental Measures in Romania; Table S2: Overview of features of the secondary dataset; Table S3: Details of machine learning processing and summary statistics. For each target, the features used in modeling and their summary statistics are presented in “Features used for modeling and summary statistics”. Metrics such as mean, median, standard deviation, parameter type (e.g., numeric or categorical), the number of unique values, and minimum and maximum values are included. A target leakage assessment is also conducted, categorizing the risk of target leakage as low, medium, or high. The “Data Quality Handling Report” outlines the processing of different feature types, methods for missing value imputation, and optimization parameters. For targets 3 and 4, the data handling reports of the individual models within the blenders are provided; Table S4: Reduced feature lists for each target.
