# Supplementary material for: AutoML-Driven Insights into Patient Outcomes and Emergency Care During Romania’s First Wave of COVID-19
Source: Bioengineering (Basel). 2024 Dec 15;11(12):1272. doi: 10.3390/bioengineering11121272 (PMC11673140; doi:10.3390/bioengineering11121272)
Supplement: Supplementary file 1 [file bioengineering-11-01272-s001.zip › Supplementary Tables S1-S4 AutoML COVID - Bioengineering final.pdf]

Table S1. COVID-19 Pandemic Governmental Measures in Romania.

Date of implementation	Randomized ID	Measures
15.05.20	37	• All patients will be considered as potentially infected with coronavirus<br>• Hair and beauty salons reopen<br>• Hotels will open, but restaurants, bars, cafes and coffee shops on hotel premises will not operate in the first phase. Meals will be served by room service, with disposable cutlery<br>• A small group of people is allowed inside churches, but not during services. These will be held outdoors
01.06.20	53	• Open-air sports competitions without spectators; open-air shows and concerts; lifting of restrictions on the movement of persons outside the locality; resumption of international rail and road transport; opening of open-air terraces; allowing access to the beach, subject to health protection measures<br>• The organization and conduct in indoor or outdoor pools of physical training activities of professional athletes and sports competitions without spectator participation; the conduct by professional athletes of physical training activities indoors<br>• The organization and staging of shows, concerts, other open-air cultural events and public and private festivals with a maximum of 500 spectators seated at least 2 meters apart<br>• The preparation, sale and consumption of food and alcoholic and non-alcoholic beverages in specially designated areas outside buildings, in the open air, with a minimum distance of 2 m between meals and with no more than 4 persons present at a meal<br>• Access of persons to the beaches only with the compulsory provision of sun loungers for each person present, ensuring a minimum distance of 2 m between sun loungers which are used by persons from different families<br>• Restrictions/ban on the movement of persons outside the locality/metropolitan area are lifted
15.06.20	60	• The quarantine/isolation measure does not apply to persons arriving from countries established on the basis of an analysis carried out by the National Institute of Public Health.<br>• Persons coming to Romania from countries covered by the exception from quarantine/isolation provided for in paragraph (1), but who, prior to their departure from that country, have not stayed for at least 14 days on its territory, shall enter quarantine/isolation at the declared residence/location, together with their roommates, including family/relatives, as the case may be.

		• Persons who have either left Romania and spend a period of less than 14 days in another country exempted from the quarantine/ isolation measure or have spent a period of at least 14 days cumulatively in countries exempted from the quarantine/ isolation measure are not quarantined/ isolated upon entry into Romania<br>• the number of persons not living together who may participate in outdoor recreational and sporting activities, i.e. cycling, hiking, running, canoeing, climbing, hunting, fishing and other outdoor recreational and sporting activities, is increased to 6<br>• the swimming activity may resume.<br>• Private events in enclosed spaces can be organized with a maximum of 20 people attending. Private events in open spaces may be organized with a maximum of 50 people attending.<br>• Retailing of goods and services is resumed in shopping centers where several economic operators are active<br>• The activity of economic operators running outdoor spas, outdoor swimming pools or sports/ fitness halls is resumed<br>• Activities are allowed to take place in nurseries, kindergartens, and after-school programs during the summer vacation period
01.07.20	12	• relaxation measures postponed
15.07.20	3	• permission to organize and conduct training sessions and group camps, in groups of up to 10 people
01.08.20	32	• Wearing a protective mask, covering both the nose and mouth, becomes mandatory for all individuals aged 5 and older, present in open public spaces, such as markets, fairs, waiting areas (bus stops, train platforms, and similar), promenades, areas where public celebrations or pilgrimages take place, and the exteriors of tourist attractions<br>• Thus, terraces, clubs, bars, etc., as well as gambling activities in the counties of Argeș, Brașov, Constanța, Dâmbovița, Ilfov, Prahova, Galați and Bucharest, were allowed to be opened only between 06.00 and 23.00
15.08.20	100	• Restaurants remained closed, as did the limitation on private event attendance to 50 people in open spaces and 20 people in closed spaces<br>• Restaurants remained closed, and the restriction limiting participation in private events to 50 people in open spaces and 20 people in closed spaces remained in place. The ban on pedestrian groups of more than 6 people not belonging to the same family within localities was maintained. The closure of children's playgrounds was also maintained
01.09.20	71	• The reopening of restaurants and cafés located inside buildings is permitted, with compliance to the protection norms established by specialists. The activity can resume in counties where the cumulative incidence of cases in the last 14 days is less than or equal to 1.5 per 1,000 inhabitants<br>• the ban on bar/ club/ disco activities is maintained<br>• reopening of cinemas and facilities for the organization of shows or concerts, subject to certain protection rules

		• a maximum of 50 people may attend private indoor events and a maximum of 100 people may attend outdoor events
14.09.20	22	• Schools start
27.09.20	52	• Local elections
01.10.20	30	• Opening of universities

Table S2. Overview of features of the secondary data set. The features "Main_Procedure", "Secondary_Procedure", "Main_Diagnosis_first", "Main_Diagnosis_second", "Secondary_Diagnoses" and "Suspected_Diagnosis" all contain ICD-10 codes. While these are recognized by the AutoML platform as text variables, they can be transformed into categorical variables, where each category represents a unique ICD-10 code. Specifically, for "Secondary_Diagnoses", which often contains multiple ICD-10 codes, a boolean transformation was performed where each ICD-10 chapter within "Secondary_Diagnoses" was converted into a standalone boolean feature indicating the presence or absence of each chapter category. The feature "Suspected_Diagnosis" represents the initial diagnosis for which a patient is admitted to the hospital, to be confirmed or ruled out during the hospital stay. "Main_Diagnosis_first" is the confirmed primary diagnosis that is the main reason for the patient's hospitalization, while "Main_Diagnosis_second" includes other primary diagnoses identified during the hospitalization that were newly discovered but were not the main reason for admission. "Secondary_Diagnoses" includes additional diagnoses identified during previous hospitalizations or outpatient visits that represent pre-existing conditions.

**Features**	**Values**
Weight_at_admission	Weight in gram
had_intensive_care	True / false
Sex	feminin/ masculin/ undetermined
Type_of_discharge_ID	Discharged / Discharged upon demand / transfer to another hospital / deceased
Main_Procedure	ICD-10 codes for main procedures
Admission_ward_ID	169 medical sections
is_deceased	True / false
Case_ID	Pseudonymized identification
Admission_Date	dd.mm.yyyy
Admission_Date_day_of_week	weekday
Admission_Date_hour_of_day	hh:mm:ss

Accident_ID	No information / Suspicion of work related accident / Suspicion of occupational disease / Traffic accident / Assault / Involuntary hospitalization authorized by Law 487/200
Admission_type_ID	without hospitalization referral / hospitalization referral from the general practitioner / hospitalization referral from a specialist / transfer to another hospital / admission on demand/ other
Ventilation_hours	Time in hours
Age	Age in years
Governmental_measures_randomized_phase_ID	Governmental interventions from 15.05./ 01.06./ 15.06./ 01.07./ 15.07./ 01.08./ 15.08./ 01.09./ 14.09./ 27.09./ 01.10.
Discharge_Date_day_of_week	weekday
NUTS2_region_of_patient	RO11/ RO12/ RO21/ RO22/ RO31/ RO32/ RO41/ RO42
Date_of_main_procedure	dd.mm.yyyy
Date_of_main_procedure_day_of_week	weekday
Date_of_main_procedure_hour_of_day	hh:mm:ss
Discharge_ward_ID	169 medical wards
has_COVID-19	True / false
Discharge_Date	dd.mm.yyyy
Discharge_Date_day_of_week	weekday
Discharge_Date_hour_of_day	hh:mm:ss
State_at_discharge_ID	Cured / ameliorated / stationary / aggravated / deceased
has_COVID-19_suspected_or_performed_test	True / false
Medical_Specialist_ID	64 medical specialties
Days_spent_in_the_intensive_care_unit	Time in days
is_cured_or_ameliorated	True / false
had_ventilation	True / false
County_administrative_region_of_patient	41 counties of Romania
Suspected_Diagnosis	ICD-10 code
Education_level_ID	no studies / elementary school / secondary school / professional school / high school / post-secondary education / short term studies / long term studies / unspecified
has_positive_test	True / false
Secondary_Procedure	ICD-10 code
Hospital_ID	Pseudonymized identification
Insurance_type_ID	National Health Insurance House / voluntary insurance / uninsured

Main_Diagnosis_second	ICD-10 code
Secondary_Diagnoses	ICD-10 code
Occupation_ID	no occupation / employee / freelancer / owner / farmer / student / unemployed / pensioner
Main_Diagnosis_first	ICD-10 code
NUTS2_region_of_hospital	RO11 / RO12 / RO21 / RO22 / RO31 / RO32 / RO41 / RO42
Admission_criteria_ID	Birth / surgical emergency with life-threatening potential / diseases with endemoepidemic potential that require isolation and treatment / obligatory hospitalization / ineffective ambulant treatment / patients requiring prolonged medical assistance
Acute_case_or_emergency	True / false
is_romanian	True / false
Citizenship_ID	247 nationalities
Secondary_Diagnoses_icd_10_chapter_12	True / false
Secondary_Diagnoses_icd_10_chapter_4	True / false
Secondary_Diagnoses_icd_10_chapter_19	True / false
Secondary_Diagnoses_icd_10_chapter_11	True / false
Secondary_Diagnoses_icd_10_chapter_20	True / false
Secondary_Diagnoses_icd_10_chapter_16	True / false
Secondary_Diagnoses_icd_10_chapter_2	True / false
Secondary_Diagnoses_icd_10_chapter_0	True / false
Secondary_Diagnoses_icd_10_chapter_8	True / false
Secondary_Diagnoses_icd_10_chapter_7	True / false
Secondary_Diagnoses_icd_10_chapter_9	True / false
Secondary_Diagnoses_icd_10_chapter_13	True / false
Secondary_Diagnoses_icd_10_chapter_22	True / false
Secondary_Diagnoses_icd_10_chapter_18	True / false
Secondary_Diagnoses_icd_10_chapter_5	True / false
Secondary_Diagnoses_icd_10_chapter_1	True / false
Secondary_Diagnoses_icd_10_chapter_6	True / false
Secondary_Diagnoses_icd_10_chapter_21	True / false
Secondary_Diagnoses_icd_10_chapter_15	True / false
Secondary_Diagnoses_icd_10_chapter_14	True / false
Secondary_Diagnoses_icd_10_chapter_10	True / false

Secondary_Diagnoses_icd_10_chapter_17	True / false
Secondary_Diagnoses_icd_10_chapter_3	True / false

Table S3. Details of machine learning processing and summary statistics. For each target, the features used in modeling and their summary statistics are presented in 'Features used for modeling and summary statistics.' Metrics such as mean, median, standard deviation (SD), parameter type (e.g., numeric or categorical), the number of unique values, and minimum and maximum values are included. A target leakage assessment is also conducted, categorizing the risk of target leakage as low, medium, or high. The 'Data Quality Handling Report' outlines the processing of different feature types, methods for missing value imputation, and optimization parameters. For targets 3 and 4, the data handling reports of the individual models within the blenders are provided.

Target 1: state at discharge: cured or ameliorated

Features for Modeling and Summary Statistics

Feature Name	Var Type	Unique	Missing	Mean	Std Dev	Median	Min	Max	Target Leakage
is_romanian	Boolean	2	0	1.0	0.045	1.0	0.0	1.0	Low
Admission_type_ID	Categorical	6	0	N/A	N/A	N/A	N/A	N/A	Low
Hospital_ID	Categorical	504	0	N/A	N/A	N/A	N/A	N/A	Low
has_positive_test	Boolean	2	0	0.09	0.29	0.0	0.0	1.0	Low
Citizenship_ID	Categorical	72	0	N/A	N/A	N/A	N/A	N/A	Low
has_covid_suspected_or_positive_test	Boolean	2	0	0.18	0.38	0.0	0.0	1.0	Low
Date_of_main_procedure	Date	40911	223235	2020-07-24T03:28:18.841630	37.69 days	2020-07-22T22:45:44.500000	2020-05-15T00:20:00	2020-09-30T15:40:00	Low
Acute_case_or_emergency	Boolean	2	0	0.93	0.26	1.0	0.0	1.0	Low

Medical_Specialist_ID	Categorical	46	3505	N/A	N/A	N/A	N/A	N/A	Low
Days_spent_in_the_intensive_care_unit	Numeric	74	0	0.37	2.0302	0.0	0.0	119.0	Low
had_ventilation	Boolean	2	0	0.02	0.14	0.0	0.0	1.0	Low
County__administrative_region__of_patient	Categorical	42	0	N/A	N/A	N/A	N/A	N/A	Low
Discharge_ward_ID	Categorical	95	0	N/A	N/A	N/A	N/A	N/A	Low
Occupation_ID	Categorical	8	27393	N/A	N/A	N/A	N/A	N/A	Low
Admission_ward_ID	Categorical	96	0	N/A	N/A	N/A	N/A	N/A	Low
NUTS2_region_of_hospital	Categorical	8	0	N/A	N/A	N/A	N/A	N/A	Low
NUTS2_region_of_patient	Categorical	8	0	N/A	N/A	N/A	N/A	N/A	Low
Insurance_type_ID	Categorical	3	0	N/A	N/A	N/A	N/A	N/A	Low
Admission_criteria_ID	Categorical	6	1	N/A	N/A	N/A	N/A	N/A	Low
had_intensive_care	Boolean	2	0	0.109	0.31	0.0	0.0	1.0	Low
Secondary_Procedure	Text	218750	14291	N/A	N/A	N/A	N/A	N/A	N/A
Main_Diagnosis__second_	Categorical	845	288091	N/A	N/A	N/A	N/A	N/A	Low
Ventilation_hours	Numeric	504	0	1.44	22.59	0.0	0.0	2720.0	Low
Age	Numeric	105	0	47.84	25.73	53.0	-7.0	108.0	Low
Accident_ID	Categorical	5	305626	N/A	N/A	N/A	N/A	N/A	Low
Main_Diagnosis__first_	Categorical	6320	0	N/A	N/A	N/A	N/A	N/A	Low
Suspected_Diagnosis	Categorical	6351	0	N/A	N/A	N/A	N/A	N/A	Low
has_covid	Categorical	2	26479	N/A	N/A	N/A	N/A	N/A	Low
governmental_measures_randomized_phase_id	Numeric	10	0	43.4	30.094	37.0	3.0	100.0	Low
Education_level_ID	Categorical	9	3745	N/A	N/A	N/A	N/A	N/A	Low

Sex	Categorical	2	0	N/A	N/A	N/A	N/A	N/A	Low
Secondary_Diagnoses_icd_10_kapitel_4_Endokrine_Ernaehrungs__und_Stoffwechselkrankheiten	Boolean	2	0	0.32	0.47	0.0	0.0	1.0	Low
Secondary_Diagnoses_icd_10_kapitel_1_Bestimmte_infektioese_und_parasitaere_Krankheiten	Boolean	2	0	0.13	0.33	0.0	0.0	1.0	Low
Secondary_Diagnoses_icd_10_kapitel_12_Krankheiten_der_Haut_und_der_Unterhaut	Boolean	2	0	0.032	0.18	0.0	0.0	1.0	Low
Secondary_Diagnoses_icd_10_kapitel_22_Schluesselnummern_fuer_besondere_Zwecke	Boolean	2	0	0.18	0.39	0.0	0.0	1.0	Low
Secondary_Diagnoses_icd_10_kapitel_6_Krankheiten_des_Nervensystems	Boolean	2	0	0.069	0.25	0.0	0.0	1.0	Low
Secondary_Diagnoses_icd_10_kapitel_8_Krankheiten_des_Ohres_und_des_Warzenfortsatzes	Boolean	2	0	0.015	0.12	0.0	0.0	1.0	Low
Secondary_Diagnoses_icd_10_kapitel_2_Neubildungen	Boolean	2	0	0.11	0.32	0.0	0.0	1.0	Low
Secondary_Diagnoses_icd_10_kapitel_11_Krankheiten_des_Verdauungssystems	Boolean	2	0	0.18	0.38	0.0	0.0	1.0	Low
Secondary_Diagnoses_icd_10_kapitel_18_Symptome_und_abnorme_klinische_und_Laborbefunde_die_anderenorts_nicht_klassifiziert_sind	Boolean	2	0	0.27	0.44	0.0	0.0	1.0	Low
Secondary_Diagnoses_icd_10_kapitel_10_Krankheiten_des_Atmungssystems	Boolean	2	0	0.16	0.37	0.0	0.0	1.0	Low
Secondary_Diagnoses_icd_10_kapitel_7_Krankheiten_des_Auges_und_der_Augenanhangsgebilde	Boolean	2	0	0.0205	0.14	0.0	0.0	1.0	Low
Secondary_Diagnoses_icd_10_kapitel_16_Bestimmte_Zustaende_die_ihren_Ursprung_in_der_Perinatalperiode_haben	Boolean	2	0	0.063	0.24	0.0	0.0	1.0	Low
Secondary_Diagnoses_icd_10_kapitel_5_Psychische_und_Verhaltensstoerungen	Boolean	2	0	0.11	0.31	0.0	0.0	1.0	Low
Secondary_Diagnoses_icd_10_kapitel_null	Boolean	1	0	1.0	0.0	1.0	1.0	1.0	N/A

Secondary_Diagnoses_icd_10_kapitel_13_Krankheiten_des_Muskel_Skelett_Systems_und_des_Bindegewebes	Boolean	2	0	0.092	0.29	0.0	0.0	1.0	Low
Secondary_Diagnoses_icd_10_kapitel_17_Angeborene_Fehlbildungen_Deformitaeten_und_Chromosomenanomalien	Boolean	2	0	0.028	0.17	0.0	0.0	1.0	Low
Secondary_Diagnoses_icd_10_kapitel_14_Krankheiten_des_Urogenitalsystems	Boolean	2	0	0.14	0.35	0.0	0.0	1.0	Low
Secondary_Diagnoses_icd_10_kapitel_15_Schwangerschaft_Geburt_und_Wochenbett	Boolean	2	0	0.092	0.29	0.0	0.0	1.0	Low
Secondary_Diagnoses_icd_10_kapitel_19_Verletzungen_Vergiftungen_und_bestimmte_andere_Folgen_aeusserer_Ursachen	Boolean	2	0	0.0605	0.24	0.0	0.0	1.0	Low
Secondary_Diagnoses_icd_10_kapitel_21_Faktoren_die_den_Gesundheitszustand_beeinflussen_und_zur_Inanspruchnahme_des_Gesundheitswesens_fuehren	Boolean	2	0	0.39	0.49	0.0	0.0	1.0	Low
Secondary_Diagnoses_icd_10_kapitel_20_Äussere_Ursachen_von_Morbiditaet_und_Mortalitaet	Boolean	2	0	0.0002	0.015	0.0	0.0	1.0	N/A
Secondary_Diagnoses_icd_10_kapitel_9_Krankheiten_des_Kreislaufsystems	Boolean	2	0	0.39	0.49	0.0	0.0	1.0	Low
Secondary_Diagnoses_icd_10_kapitel_3_Krankheiten_des_Blutes_und_der_blutbildenden_Organe_sowie_bestimmte_Stoerungen_mit_Beteiligung_des_Immunsystems	Boolean	2	0	0.17	0.37	0.0	0.0	1.0	Low

Data Quality Handling Report

Feature Name	Var Type	Missing Count	Missing Percentage	Imputation Name	Imputation Description
Accident_ID	Categorical	359405	99	Ordinal encoding of categorical variables	Imputed value: -2
Main_Diagnosis__second_	Categorical	339054	94	Ordinal encoding of categorical variables	Imputed value: -2
Date_of_main_procedure (Day of Week)	Categorical	262550	73	Ordinal encoding of categorical variables	Imputed value: -2
Date_of_main_procedure (Hour of Day)	Categorical	262550	73	Ordinal encoding of categorical variables	Imputed value: -2
Date_of_main_procedure	Numeric	262550	73	Missing Values Imputed	Imputed value: -9999
Occupation_ID	Categorical	32350	9	Ordinal encoding of categorical variables	Imputed value: -2
has_covid	Categorical	31047	9	Ordinal encoding of categorical variables	Imputed value: -2
Education_level_ID	Categorical	4398	1	Ordinal encoding of categorical variables	Imputed value: -2

Medical_Specialist_ID	Categorical	4208	1	Ordinal encoding of categorical variables	Imputed value: -2
Admission_criteria_ID	Categorical	1	0	Ordinal encoding of categorical variables	Imputed value: -2
Admission_type_ID	Categorical	0	0	Ordinal encoding of categorical variables	Imputed value: -2
Hospital_ID	Categorical	0	0	Ordinal encoding of categorical variables	Imputed value: -2
Citizenship_ID	Categorical	0	0	Ordinal encoding of categorical variables	Imputed value: -2
County__administrative_region__of_patient	Categorical	0	0	Ordinal encoding of categorical variables	Imputed value: -2
Discharge_ward_ID	Categorical	0	0	Ordinal encoding of categorical variables	Imputed value: -2
Admission_ward_ID	Categorical	0	0	Ordinal encoding of categorical variables	Imputed value: -2
NUTS2_region_of_hospital	Categorical	0	0	Ordinal encoding of categorical variables	Imputed value: -2

NUTS2_region_of_patient	Categorical	0	0	Ordinal encoding of categorical variables	Imputed value: -2
Insurance_type_ID	Categorical	0	0	Ordinal encoding of categorical variables	Imputed value: -2
Main_Diagnosis__first_	Categorical	0	0	Ordinal encoding of categorical variables	Imputed value: -2
Suspected_Diagnosis	Categorical	0	0	Ordinal encoding of categorical variables	Imputed value: -2
Sex	Categorical	0	0	Ordinal encoding of categorical variables	Imputed value: -2
governmental_measures_randomized_phase_id (Categorical Int)	Categorical	0	0	Ordinal encoding of categorical variables	Imputed value: -2
is_romanian	Numeric	0	0	Missing Values Imputed	Imputed value: 1
has_positive_test	Numeric	0	0	Missing Values Imputed	Imputed value: 0
has_covid_suspected_or_positive_test	Numeric	0	0	Missing Values Imputed	Imputed value: 0
Acute_case_or_emergency	Numeric	0	0	Missing Values Imputed	Imputed value: 1

Days_spent_in_the_intensive_care_unit	Numeric	0	0	Missing Values Imputed	Imputed value: 0
had_ventilation	Numeric	0	0	Missing Values Imputed	Imputed value: 0
had_intensive_care	Numeric	0	0	Missing Values Imputed	Imputed value: 0
Ventilation_hours	Numeric	0	0	Missing Values Imputed	Imputed value: 0
Age	Numeric	0	0	Missing Values Imputed	Imputed value: 53
Secondary_Diagnoses_icd_10_kapitel_4_Endokrine_Ernaehrungs__und_Stoffwechselkrankheiten	Numeric	0	0	Missing Values Imputed	Imputed value: 0
Secondary_Diagnoses_icd_10_kapitel_1_Bestimmte_infektioese_und_parasitaere_Krankheiten	Numeric	0	0	Missing Values Imputed	Imputed value: 0
Secondary_Diagnoses_icd_10_kapitel_12_Krankheiten_der_Haut_und_der_Unterhaut	Numeric	0	0	Missing Values Imputed	Imputed value: 0
Secondary_Diagnoses_icd_10_kapitel_22_Schluesselnummern_fuer_besondere_Zwecke	Numeric	0	0	Missing Values Imputed	Imputed value: 0
Secondary_Diagnoses_icd_10_kapitel_6_Krankheiten_des_Nervensystems	Numeric	0	0	Missing Values Imputed	Imputed value: 0
Secondary_Diagnoses_icd_10_kapitel_8_Krankheiten_des_Ohres_und_des_Warzenfortsatzes	Numeric	0	0	Missing Values Imputed	Imputed value: 0

Secondary_Diagnoses_icd_10_kapitel_2_Neubildungen	Numeric	0	0	Missing Values Imputed	Imputed value: 0
Secondary_Diagnoses_icd_10_kapitel_11_Krankheiten_des_Verdauungssystems	Numeric	0	0	Missing Values Imputed	Imputed value: 0
Secondary_Diagnoses_icd_10_kapitel_18_Symptome_und_abnorme_klinische_und_Laborbefunde_die_anderenorts_nicht_klassifiziert_sind	Numeric	0	0	Missing Values Imputed	Imputed value: 0
Secondary_Diagnoses_icd_10_kapitel_10_Krankheiten_des_Atmungssystems	Numeric	0	0	Missing Values Imputed	Imputed value: 0
Secondary_Diagnoses_icd_10_kapitel_7_Krankheiten_des_Auges_und_der_Augenanhangsgebilde	Numeric	0	0	Missing Values Imputed	Imputed value: 0
Secondary_Diagnoses_icd_10_kapitel_16_Bestimmte_Zustaende_die_ihren_Ursprung_in_der_Perinatalperiode_haben	Numeric	0	0	Missing Values Imputed	Imputed value: 0
Secondary_Diagnoses_icd_10_kapitel_5_Psychische_und_Verhaltensstoerungen	Numeric	0	0	Missing Values Imputed	Imputed value: 0
Secondary_Diagnoses_icd_10_kapitel_null	Numeric	0	0	Missing Values Imputed	Imputed value: 0
Secondary_Diagnoses_icd_10_kapitel_13_Krankheiten_des_Muskel_Skelett_Systems_und_des_Bindegewebes	Numeric	0	0	Missing Values Imputed	Imputed value: 0
Secondary_Diagnoses_icd_10_kapitel_17_Angeborene_Fehlbildungen_Deformitaeten_und_Chromosomenanomalien	Numeric	0	0	Missing Values Imputed	Imputed value: 0
Secondary_Diagnoses_icd_10_kapitel_14_Krankheiten_des_Urogenitalsystems	Numeric	0	0	Missing Values Imputed	Imputed value: 0

Secondary_Diagnoses_icd_10_kapitel_15_Schwangerschaft_Geburt_und_Wochenbett	Numeric	0	0	Missing Values Imputed	Imputed value: 0
Secondary_Diagnoses_icd_10_kapitel_19_Verletzungen_Vergiftungen_und_bestimmte_andere_Folgen_aeusserer_Ursachen	Numeric	0	0	Missing Values Imputed	Imputed value: 0
Secondary_Diagnoses_icd_10_kapitel_21_Faktoren_die_den_Gesundheitszustand_beeinflussen_und_zur_Inanspruchnahme_des_Gesundheitswesens_fuehren	Numeric	0	0	Missing Values Imputed	Imputed value: 0
Secondary_Diagnoses_icd_10_kapitel_20_Äussere_Ursachen_von_Morbiditaet_und_Mortalitaet	Numeric	0	0	Missing Values Imputed	Imputed value: 0
Secondary_Diagnoses_icd_10_kapitel_9_Krankheiten_des_Kreislaufsystems	Numeric	0	0	Missing Values Imputed	Imputed value: 0
Secondary_Diagnoses_icd_10_kapitel_3_Krankheiten_des_Blutes_und_der_blutbildenden_Organe_sowie_bestimmte_Stoerungen_mit_Beteiligung_des_Immunsystems	Numeric	0	0	Missing Values Imputed	Imputed value: 0

Target 2: state at discharge: deceased

Features for Modeling and Summary Statistics

Feature Name	Var Type	Unique	Missing	Mean	Std Dev	Median	Min	Max	Target Leakage
Main_Procedure	Categorical	2480	218898	N/A	N/A	N/A	N/A	N/A	Low
is_romanian	Boolean	2	0	1.0	0.044	1.0	0.0	1.0	Low
Admission_type_ID	Categorical	6	0	N/A	N/A	N/A	N/A	N/A	Low
Hospital_ID	Categorical	502	0	N/A	N/A	N/A	N/A	N/A	Low
has_positive_test	Boolean	2	0	0.086	0.28	0.0	0.0	1.0	Low
Citizenship_ID	Categorical	66	0	N/A	N/A	N/A	N/A	N/A	Low
has_covid_suspected_or_positive_test	Boolean	2	0	0.18	0.38	0.0	0.0	1.0	Low
Date_of_main_procedure	Date	42026	218903	2020-07-24T01:46:22.558687	37.74 days	2020-07-22T14:20:00	2020-05-15T00:20:00	2020-09-30T15:30:00	Low
Acute_case_or_emergency	Boolean	2	0	0.93	0.26	1.0	0.0	1.0	Low
Medical_Specialist_ID	Categorical	47	3482	N/A	N/A	N/A	N/A	N/A	Low
Days_spent_in_the_intensive_care_unit	Numeric	77	0	0.37	2.055	0.0	-1.0	91.0	Low
had_ventilation	Boolean	2	0	0.0202	0.14	0.0	0.0	1.0	Low
County__administrative_region__of_patient	Categorical	42	0	N/A	N/A	N/A	N/A	N/A	Low

Discharge_ward_ID	Categorical	94	0	N/A	N/A	N/A	N/A	N/A	Low
Occupation_ID	Categorical	8	27512	N/A	N/A	N/A	N/A	N/A	Low
Admission_ward_ID	Categorical	96	0	N/A	N/A	N/A	N/A	N/A	Low
NUTS2_region_of_hospital	Categorical	8	0	N/A	N/A	N/A	N/A	N/A	Low
NUTS2_region_of_patient	Categorical	8	0	N/A	N/A	N/A	N/A	N/A	Low
Insurance_type_ID	Categorical	3	0	N/A	N/A	N/A	N/A	N/A	Low
Admission_criteria_ID	Categorical	6	1	N/A	N/A	N/A	N/A	N/A	Low
had_intensive_care	Boolean	2	0	0.11	0.31	0.0	0.0	1.0	Low
Secondary_Procedure	Text	218850	13462	N/A	N/A	N/A	N/A	N/A	N/A
Main_Diagnosis__second_	Categorical	842	286216	N/A	N/A	N/A	N/A	N/A	Low
Ventilation_hours	Numeric	517	0	1.46	23.36	0.0	0.0	3940.0	Low
Age	Numeric	104	0	47.54	25.84	53.0	0.0	108.0	Low
Accident_ID	Categorical	5	304020	N/A	N/A	N/A	N/A	N/A	Low
Suspected_Diagnosis	Categorical	6314	0	N/A	N/A	N/A	N/A	N/A	Low
has_covid	Categorical	2	26738	N/A	N/A	N/A	N/A	N/A	Low
governmental_measures_randomized_phase_id	Numeric	10	0	43.41	30.071	37.0	3.0	100.0	Low
Education_level_ID	Categorical	9	3598	N/A	N/A	N/A	N/A	N/A	Low

Sex	Categorical	2	0	N/A	N/A	N/A	N/A	N/A	Low
Secondary_Diagnoses_icd_10_kapitel_4_Endokrine_Ernaehrungs__und_Stoffwechselkrankheiten	Boolean	2	0	0.32	0.47	0.0	0.0	1.0	Low
Secondary_Diagnoses_icd_10_kapitel_1_Bestimmte_infektioese_und_parasitaere_Krankheiten	Boolean	2	0	0.12	0.33	0.0	0.0	1.0	Low
Secondary_Diagnoses_icd_10_kapitel_12_Krankheiten_der_Haut_und_der_Unterhaut	Boolean	2	0	0.032	0.18	0.0	0.0	1.0	Low
Secondary_Diagnoses_icd_10_kapitel_22_Schluesselnummern_fuer_besondere_Zwecke	Boolean	2	0	0.18	0.38	0.0	0.0	1.0	Low
Secondary_Diagnoses_icd_10_kapitel_6_Krankheiten_des_Nervensystems	Boolean	2	0	0.069	0.25	0.0	0.0	1.0	Low
Secondary_Diagnoses_icd_10_kapitel_8_Krankheiten_des_Ohres_und_des_Warzenfortsatzes	Boolean	2	0	0.015	0.12	0.0	0.0	1.0	Low
Secondary_Diagnoses_icd_10_kapitel_2_Neubildungen	Boolean	2	0	0.105	0.307	0.0	0.0	1.0	Low
Secondary_Diagnoses_icd_10_kapitel_11_Krankheiten_des_Verdauungssystems	Boolean	2	0	0.18	0.39	0.0	0.0	1.0	Low
Secondary_Diagnoses_icd_10_kapitel_18_Symptome_und_abnorme_klinische_und_Laborbefunde_die_anderenorts_nicht_klassifiziert_sind	Boolean	2	0	0.27	0.44	0.0	0.0	1.0	Low
Secondary_Diagnoses_icd_10_kapitel_10_Krankheiten_des_Atmungssystems	Boolean	2	0	0.16	0.37	0.0	0.0	1.0	Low
Secondary_Diagnoses_icd_10_kapitel_7_Krankheiten_des_Auges_und_der_Augenanhangsgebilde	Boolean	2	0	0.0207	0.14	0.0	0.0	1.0	Low
Secondary_Diagnoses_icd_10_kapitel_16_Bestimmte_Zustaende_die_ihren_Ursprung_in_der_Perinatalperiode_haben	Boolean	2	0	0.065	0.25	0.0	0.0	1.0	Low
Secondary_Diagnoses_icd_10_kapitel_5_Psychische_und_Verhaltensstoerungen	Boolean	2	0	0.108	0.31	0.0	0.0	1.0	Low
Secondary_Diagnoses_icd_10_kapitel_null	Boolean	1	0	1.0	0.0	1.0	1.0	1.0	N/A

Secondary_Diagnoses_icd_10_kapitel_13_Krankheiten_des_Muskel_Skelett_Systems_und_des_Bindegewebes	Boolean	2	0	0.093	0.29	0.0	0.0	1.0	Low
Secondary_Diagnoses_icd_10_kapitel_17_Angeborene_Fehlbildungen_Deformitaeten_und_Chromosomenanomalien	Boolean	2	0	0.028	0.17	0.0	0.0	1.0	Low
Secondary_Diagnoses_icd_10_kapitel_14_Krankheiten_des_Urogenitalsystems	Boolean	2	0	0.14	0.35	0.0	0.0	1.0	Low
Secondary_Diagnoses_icd_10_kapitel_15_Schwangerschaft_Geburt_und_Wochenbett	Boolean	2	0	0.095	0.29	0.0	0.0	1.0	Low
Secondary_Diagnoses_icd_10_kapitel_19_Verletzungen_Vergiftungen_und_bestimmte_andere_Folgen_aeusserer_Ursachen	Boolean	2	0	0.061	0.24	0.0	0.0	1.0	Low
Secondary_Diagnoses_icd_10_kapitel_21_Faktoren_die_den_Gesundheitszustand_beeinflussen_und_zur_Inanspruchnahme_des_Gesundheitswesens_fuehren	Boolean	2	0	0.39	0.49	0.0	0.0	1.0	Low
Secondary_Diagnoses_icd_10_kapitel_20_Äussere_Ursachen_von_Morbiditaet_und_Mortalitaet	Boolean	2	0	0.0002	0.015	0.0	0.0	1.0	N/A
Secondary_Diagnoses_icd_10_kapitel_9_Krankheiten_des_Kreislaufsystems	Boolean	2	0	0.39	0.49	0.0	0.0	1.0	Low
Secondary_Diagnoses_icd_10_kapitel_3_Krankheiten_des_Blutes_und_der_blutbildenden_Organe_sowie_bestimmte_Stoerungen_mit_Beteiligung_des_Immunsystems	Boolean	2	0	0.17	0.37	0.0	0.0	1.0	Low

Data Quality Handling Report

Feature Name	Var Type	Missing Count	Missing Percentage	Imputation Name	Imputation Description
Accident_ID	Categorical	357678	99	One-Hot Encoding	Missing indicator treated as feature
Accident_ID	Categorical	357678	99	Ordinal encoding of categorical variables	Imputed value: -2
Main_Diagnosis__second_	Categorical	336615	93	One-Hot Encoding	Missing indicator treated as feature
Main_Diagnosis__second_	Categorical	336615	93	Ordinal encoding of categorical variables	Imputed value: -2
Date_of_main_procedure (Day of Week)	Categorical	257484	72	One-Hot Encoding	Missing indicator treated as feature
Date_of_main_procedure (Day of Week)	Categorical	257484	72	Ordinal encoding of categorical variables	Imputed value: -2
Date_of_main_procedure (Hour of Day)	Categorical	257484	72	One-Hot Encoding	Missing indicator treated as feature
Date_of_main_procedure (Hour of Day)	Categorical	257484	72	Ordinal encoding of categorical variables	Imputed value: -2
Date_of_main_procedure	Numeric	257484	72	Missing Values Imputed	Missing indicator treated as feature, Imputed value: 1.5954318e+12
Main_Procedure	Categorical	257477	72	One-Hot Encoding	Missing indicator treated as feature

Main_Procedure	Categorical	257477	72	Ordinal encoding of categorical variables	Imputed value: -2
Occupation_ID	Categorical	32483	9	One-Hot Encoding	Missing indicator treated as feature
Occupation_ID	Categorical	32483	9	Ordinal encoding of categorical variables	Imputed value: -2
has_covid	Categorical	31379	9	One-Hot Encoding	Missing indicator treated as feature
has_covid	Categorical	31379	9	Ordinal encoding of categorical variables	Imputed value: -2
Education_level_ID	Categorical	4207	1	One-Hot Encoding	Missing indicator treated as feature
Education_level_ID	Categorical	4207	1	Ordinal encoding of categorical variables	Imputed value: -2
Medical_Specialist_ID	Categorical	4156	1	One-Hot Encoding	Missing indicator treated as feature
Medical_Specialist_ID	Categorical	4156	1	Ordinal encoding of categorical variables	Imputed value: -2
Admission_criteria_ID	Categorical	1	0	One-Hot Encoding	Missing values treated as infrequent
Admission_criteria_ID	Categorical	1	0	Ordinal encoding of categorical variables	Imputed value: -2

Admission_type_ID	Categorical	0	0	One-Hot Encoding	Missing values ignored
Admission_type_ID	Categorical	0	0	Ordinal encoding of categorical variables	Imputed value: -2
Hospital_ID	Categorical	0	0	One-Hot Encoding	Missing values treated as infrequent
Hospital_ID	Categorical	0	0	Ordinal encoding of categorical variables	Imputed value: -2
Citizenship_ID	Categorical	0	0	One-Hot Encoding	Missing values treated as infrequent
Citizenship_ID	Categorical	0	0	Ordinal encoding of categorical variables	Imputed value: -2
County__administrative_region__of_patient	Categorical	0	0	One-Hot Encoding	Missing values ignored
County__administrative_region__of_patient	Categorical	0	0	Ordinal encoding of categorical variables	Imputed value: -2
Discharge_ward_ID	Categorical	0	0	One-Hot Encoding	Missing values treated as infrequent
Discharge_ward_ID	Categorical	0	0	Ordinal encoding of categorical variables	Imputed value: -2
Admission_ward_ID	Categorical	0	0	One-Hot Encoding	Missing values treated as infrequent

Admission_ward_ID	Categorical	0	0	Ordinal encoding of categorical variables	Imputed value: -2
NUTS2_region_of_hospital	Categorical	0	0	One-Hot Encoding	Missing values ignored
NUTS2_region_of_hospital	Categorical	0	0	Ordinal encoding of categorical variables	Imputed value: -2
NUTS2_region_of_patient	Categorical	0	0	One-Hot Encoding	Missing values ignored
NUTS2_region_of_patient	Categorical	0	0	Ordinal encoding of categorical variables	Imputed value: -2
Insurance_type_ID	Categorical	0	0	One-Hot Encoding	Missing values ignored
Insurance_type_ID	Categorical	0	0	Ordinal encoding of categorical variables	Imputed value: -2
Suspected_Diagnosis	Categorical	0	0	One-Hot Encoding	Missing values treated as infrequent
Suspected_Diagnosis	Categorical	0	0	Ordinal encoding of categorical variables	Imputed value: -2
Sex	Categorical	0	0	One-Hot Encoding	Missing values ignored
Sex	Categorical	0	0	Ordinal encoding of categorical variables	Imputed value: -2

governmental_measures_randomized_phase_id (Categorical Int)	Categorical	0	0	One-Hot Encoding	Missing values ignored
governmental_measures_randomized_phase_id (Categorical Int)	Categorical	0	0	Ordinal encoding of categorical variables	Imputed value: -2
is_romanian	Numeric	0	0	Missing Values Imputed	Imputed value: 1
has_positive_test	Numeric	0	0	Missing Values Imputed	Imputed value: 0
has_covid_suspected_or_positive_test	Numeric	0	0	Missing Values Imputed	Imputed value: 0
Acute_case_or_emergency	Numeric	0	0	Missing Values Imputed	Imputed value: 1
Days_spent_in_the_intensive_care_unit	Numeric	0	0	Missing Values Imputed	Imputed value: 0
had_ventilation	Numeric	0	0	Missing Values Imputed	Imputed value: 0
had_intensive_care	Numeric	0	0	Missing Values Imputed	Imputed value: 0
Ventilation_hours	Numeric	0	0	Missing Values Imputed	Imputed value: 0
Age	Numeric	0	0	Missing Values Imputed	Imputed value: 53

Secondary_Diagnoses_icd_10_kapitel_4_Endokrine_Ernaehrungs__und_Stoffwechselkrankheiten	Numeric	0	0	Missing Values Imputed	Imputed value: 0
Secondary_Diagnoses_icd_10_kapitel_1_Bestimmte_infektioese_und_parasitaere_Krankheiten	Numeric	0	0	Missing Values Imputed	Imputed value: 0
Secondary_Diagnoses_icd_10_kapitel_12_Krankheiten_der_Haut_und_der_Unterhaut	Numeric	0	0	Missing Values Imputed	Imputed value: 0
Secondary_Diagnoses_icd_10_kapitel_22_Schluesselnummern_fuer_besondere_Zwecke	Numeric	0	0	Missing Values Imputed	Imputed value: 0
Secondary_Diagnoses_icd_10_kapitel_6_Krankheiten_des_Nervensystems	Numeric	0	0	Missing Values Imputed	Imputed value: 0
Secondary_Diagnoses_icd_10_kapitel_8_Krankheiten_des_Ohres_und_des_Warzenfortsatzes	Numeric	0	0	Missing Values Imputed	Imputed value: 0
Secondary_Diagnoses_icd_10_kapitel_2_Neubildungen	Numeric	0	0	Missing Values Imputed	Imputed value: 0
Secondary_Diagnoses_icd_10_kapitel_11_Krankheiten_des_Verdauungssystems	Numeric	0	0	Missing Values Imputed	Imputed value: 0
Secondary_Diagnoses_icd_10_kapitel_18_Symptome_und_abnorme_klinische_und_Laborbefunde_die_anderenorts_nicht_klassifiziert_sind	Numeric	0	0	Missing Values Imputed	Imputed value: 0
Secondary_Diagnoses_icd_10_kapitel_10_Krankheiten_des_Atmungssystems	Numeric	0	0	Missing Values Imputed	Imputed value: 0
Secondary_Diagnoses_icd_10_kapitel_7_Krankheiten_des_Auges_und_der_Augenanhangsgebilde	Numeric	0	0	Missing Values Imputed	Imputed value: 0

Secondary_Diagnoses_icd_10_kapitel_16_Bestimmte_Zustaende_die_ihren_Ursprung_in_der_Perinatalperiode_haben	Numeric	0	0	Missing Values Imputed	Imputed value: 0
Secondary_Diagnoses_icd_10_kapitel_5_Psychische_und_Verhaltensstoerungen	Numeric	0	0	Missing Values Imputed	Imputed value: 0
Secondary_Diagnoses_icd_10_kapitel_null	Numeric	0	0	Missing Values Imputed	Imputed value: 0
Secondary_Diagnoses_icd_10_kapitel_13_Krankheiten_des_Muskel_Skelett_Systems_und_des_Bindegewebes	Numeric	0	0	Missing Values Imputed	Imputed value: 0
Secondary_Diagnoses_icd_10_kapitel_17_Angeborene_Fehlbildungen_Deformitaeten_und_Chromosomenanomalien	Numeric	0	0	Missing Values Imputed	Imputed value: 0
Secondary_Diagnoses_icd_10_kapitel_14_Krankheiten_des_Urogenitalsystems	Numeric	0	0	Missing Values Imputed	Imputed value: 0
Secondary_Diagnoses_icd_10_kapitel_15_Schwangerschaft_Geburt_und_Wochenbett	Numeric	0	0	Missing Values Imputed	Imputed value: 0
Secondary_Diagnoses_icd_10_kapitel_19_Verletzungen_Vergiftungen_und_bestimmte_andere_Folgen_aeusserer_Ursachen	Numeric	0	0	Missing Values Imputed	Imputed value: 0
Secondary_Diagnoses_icd_10_kapitel_21_Faktoren_die_den_Gesundheitszustand_beeinflussen_und_zur_Inanspruchnahme_des_Gesundheitswesens_fuehren	Numeric	0	0	Missing Values Imputed	Imputed value: 0
Secondary_Diagnoses_icd_10_kapitel_20_Äussere_Ursachen_von_Morbiditaet_und_Mortalitaet	Numeric	0	0	Missing Values Imputed	Imputed value: 0
Secondary_Diagnoses_icd_10_kapitel_9_Krankheiten_des_Kreislaufsystems	Numeric	0	0	Missing Values Imputed	Imputed value: 0

Secondary_Diagnoses_icd_10_kapitel_3_Krankheiten_des_Blutes_und_der_blut bildenden_Organe_sowie_bestimmte_Stoerungen_mit_Beteiligung_des_Immun systems	Numeric	0	0	Missing Values Imputed	Imputed value: 0

Target 3: acute case or emergency

Features for Modeling and Summary Statistics

Feature Name	Var Type	Unique	Missing	Mean	Std Dev	Median	Min	Max	Target Leakage
is_romanian	Boolean	2	0	1.0	0.044	1.0	0.0	1.0	Low
Admission_type_ID	Categorical	6	0	N/A	N/A	N/A	N/A	N/A	Low
has_positive_test	Boolean	2	0	0.084	0.28	0.0	0.0	1.0	Low
Citizenship_ID	Categorical	69	0	N/A	N/A	N/A	N/A	N/A	Low
has_covid_suspected_or_positive_test	Boolean	2	0	0.17	0.38	0.0	0.0	1.0	Low
County__administrative_region__of_patient	Categorical	42	0	N/A	N/A	N/A	N/A	N/A	Low
NUTS2_region_of_hospital	Categorical	8	0	N/A	N/A	N/A	N/A	N/A	Low
NUTS2_region_of_patient	Categorical	8	0	N/A	N/A	N/A	N/A	N/A	Low
Insurance_type_ID	Categorical	3	0	N/A	N/A	N/A	N/A	N/A	Low
Secondary_Procedure	Text	216559	14591	N/A	N/A	N/A	N/A	N/A	N/A
Age	Numeric	105	0	47.52	25.78	53.0	-7.0	108.0	Low
Accident_ID	Categorical	5	305388	N/A	N/A	N/A	N/A	N/A	Low
governmental_measures_randomized_phase_id	Numeric	10	0	43.34	30.088	37.0	3.0	100.0	Low

Education_level_ID	Categorical	9	4013	N/A	N/A	N/A	N/A	N/A	Low
Sex	Categorical	2	0	N/A	N/A	N/A	N/A	N/A	Low
Secondary_Diagnoses_icd_10_kapitel_4_Endokrine_Ernaehrungs__und_Stoffwechselkrankheiten	Boolean	2	0	0.32	0.47	0.0	0.0	1.0	Low
Secondary_Diagnoses_icd_10_kapitel_1_Bestimmte_infektioese_und_parasitaere_Krankheiten	Boolean	2	0	0.12	0.33	0.0	0.0	1.0	Low
Secondary_Diagnoses_icd_10_kapitel_12_Krankheiten_der_Haut_und_der_Unterhaut	Boolean	2	0	0.032	0.18	0.0	0.0	1.0	Low
Secondary_Diagnoses_icd_10_kapitel_22_Schluesselnummern_fuer_besondere_Zwecke	Boolean	2	0	0.17	0.38	0.0	0.0	1.0	Low
Secondary_Diagnoses_icd_10_kapitel_6_Krankheiten_des_Nervensystems	Boolean	2	0	0.069	0.25	0.0	0.0	1.0	Low
Secondary_Diagnoses_icd_10_kapitel_8_Krankheiten_des_Ohres_und_des_Warzenfortsatzes	Boolean	2	0	0.016	0.12	0.0	0.0	1.0	Low
Secondary_Diagnoses_icd_10_kapitel_2_Neubildungen	Boolean	2	0	0.102	0.303	0.0	0.0	1.0	Low
Secondary_Diagnoses_icd_10_kapitel_11_Krankheiten_des_Verdauungssystems	Boolean	2	0	0.18	0.38	0.0	0.0	1.0	Low
Secondary_Diagnoses_icd_10_kapitel_18_Symptome_und_abnorme_klinische_und_Laborbefunde_die_anderenorts_nicht_klassifiziert_sind	Boolean	2	0	0.25	0.43	0.0	0.0	1.0	Low
Secondary_Diagnoses_icd_10_kapitel_10_Krankheiten_des_Atmungssystems	Boolean	2	0	0.15	0.36	0.0	0.0	1.0	Low
Secondary_Diagnoses_icd_10_kapitel_7_Krankheiten_des_Auges_und_der_Augenanhangsgebilde	Boolean	2	0	0.0205	0.14	0.0	0.0	1.0	Low
Secondary_Diagnoses_icd_10_kapitel_16_Bestimmte_Zustaende_die_ihren_Ursprung_in_der_Perinatalperiode_haben	Boolean	2	0	0.066	0.25	0.0	0.0	1.0	Low
Secondary_Diagnoses_icd_10_kapitel_5_Psychische_und_Verhaltensstoerungen	Boolean	2	0	0.11	0.31	0.0	0.0	1.0	Low

Secondary_Diagnoses_icd_10_kapitel_null	Boolean	1	0	1.0	0.0	1.0	1.0	1.0	N/A
Secondary_Diagnoses_icd_10_kapitel_13_Krankheiten_des_Muskel_Skelett_Systems_und_des_Bindegewebes	Boolean	2	0	0.104	0.305	0.0	0.0	1.0	Low
Secondary_Diagnoses_icd_10_kapitel_17_Angeborene_Fehlbildungen_Deformitaeten_und_Chromosomenanomalien	Boolean	2	0	0.029	0.17	0.0	0.0	1.0	Low
Secondary_Diagnoses_icd_10_kapitel_14_Krankheiten_des_Urogenitalsystems	Boolean	2	0	0.14	0.34	0.0	0.0	1.0	Low
Secondary_Diagnoses_icd_10_kapitel_15_Schwangerschaft_Geburt_und_Wochenbett	Boolean	2	0	0.092	0.29	0.0	0.0	1.0	Low
Secondary_Diagnoses_icd_10_kapitel_19_Verletzungen_Vergiftungen_und_bestimmte_andere_Folgen_aeusserer_Ursachen	Boolean	2	0	0.0601	0.24	0.0	0.0	1.0	Low
Secondary_Diagnoses_icd_10_kapitel_21_Faktoren_die_den_Gesundheitszustand_beeinflussen_und_zur_Inanspruchnahme_des_Gesundheitswesens_fuehren	Boolean	2	0	0.38	0.49	0.0	0.0	1.0	Low
Secondary_Diagnoses_icd_10_kapitel_20_Äussere_Ursachen_von_Morbiditaet_und_Mortalitaet	Boolean	2	0	0.0002	0.014	0.0	0.0	1.0	N/A
Secondary_Diagnoses_icd_10_kapitel_9_Krankheiten_des_Kreislaufsystems	Boolean	2	0	0.38	0.49	0.0	0.0	1.0	Low
Secondary_Diagnoses_icd_10_kapitel_3_Krankheiten_des_Blutes_und_der_blutbildenden_Organe_sowie_bestimmte_Stoerungen_mit_Beteiligung_des_Immunsystems	Boolean	2	0	0.16	0.37	0.0	0.0	1.0	Low

Data Quality Handling Report

Light Gradient Boosting on ElasticNet Predictions

Feature Name	Var Type	Missing Count	Missing Percentage	Imputation Name	Imputation Description
Accident_ID	Categorical	359107	99	One-Hot Encoding	Missing indicator treated as feature
Accident_ID	Categorical	359107	99	Ordinal encoding of categorical variables	Imputed value: -2
Education_level_ID	Categorical	4646	1	One-Hot Encoding	Missing indicator treated as feature
Education_level_ID	Categorical	4646	1	Ordinal encoding of categorical variables	Imputed value: -2
Admission_type_ID	Categorical	0	0	One-Hot Encoding	Missing values ignored
Admission_type_ID	Categorical	0	0	Ordinal encoding of categorical variables	Imputed value: -2
Citizenship_ID	Categorical	0	0	One-Hot Encoding	Missing values treated as infrequent
Citizenship_ID	Categorical	0	0	Ordinal encoding of categorical variables	Imputed value: -2

County__administrative_region__of_patient	Categorical	0	0	One-Hot Encoding	Missing values ignored
County__administrative_region__of_patient	Categorical	0	0	Ordinal encoding of categorical variables	Imputed value: -2
NUTS2_region_of_hospital	Categorical	0	0	One-Hot Encoding	Missing values ignored
NUTS2_region_of_hospital	Categorical	0	0	Ordinal encoding of categorical variables	Imputed value: -2
NUTS2_region_of_patient	Categorical	0	0	One-Hot Encoding	Missing values ignored
NUTS2_region_of_patient	Categorical	0	0	Ordinal encoding of categorical variables	Imputed value: -2
Insurance_type_ID	Categorical	0	0	One-Hot Encoding	Missing values ignored
Insurance_type_ID	Categorical	0	0	Ordinal encoding of categorical variables	Imputed value: -2
Sex	Categorical	0	0	One-Hot Encoding	Missing values ignored
Sex	Categorical	0	0	Ordinal encoding of categorical variables	Imputed value: -2

governmental_measures_randomized_phase_id (Categorical Int)	Categorical	0	0	One-Hot Encoding	Missing values ignored
governmental_measures_randomized_phase_id (Categorical Int)	Categorical	0	0	Ordinal encoding of categorical variables	Imputed value: -2
is_romanian	Numeric	0	0	Missing Values Imputed	Imputed value: 1
has_positive_test	Numeric	0	0	Missing Values Imputed	Imputed value: 0
has_covid_suspected_or_positive_test	Numeric	0	0	Missing Values Imputed	Imputed value: 0
Age	Numeric	0	0	Missing Values Imputed	Imputed value: 53
Secondary_Diagnoses_icd_10_kapitel_4_Endokrine_Ernaehrungs__und_Stoffwechselkrankheiten	Numeric	0	0	Missing Values Imputed	Imputed value: 0
Secondary_Diagnoses_icd_10_kapitel_1_Bestimmte_infektioese_und_parasitaere_Krankheiten	Numeric	0	0	Missing Values Imputed	Imputed value: 0
Secondary_Diagnoses_icd_10_kapitel_12_Krankheiten_der_Haut_und_der_Unterhaut	Numeric	0	0	Missing Values Imputed	Imputed value: 0
Secondary_Diagnoses_icd_10_kapitel_22_Schluesselnummern_fuer_besondere_Zwecke	Numeric	0	0	Missing Values Imputed	Imputed value: 0
Secondary_Diagnoses_icd_10_kapitel_6_Krankheiten_des_Nervensystems	Numeric	0	0	Missing Values Imputed	Imputed value: 0

Secondary_Diagnoses_icd_10_kapitel_8_Krankheiten_des_Ohres_und_des_Warzenfortsatzes	Numeric	0	0	Missing Values Imputed	Imputed value: 0
Secondary_Diagnoses_icd_10_kapitel_2_Neubildungen	Numeric	0	0	Missing Values Imputed	Imputed value: 0
Secondary_Diagnoses_icd_10_kapitel_11_Krankheiten_des_Verdauungssystems	Numeric	0	0	Missing Values Imputed	Imputed value: 0
Secondary_Diagnoses_icd_10_kapitel_18_Symptome_und_abnorme_klinische_und_Laborbefunde_die_anderenorts_nicht_klassifiziert_sind	Numeric	0	0	Missing Values Imputed	Imputed value: 0
Secondary_Diagnoses_icd_10_kapitel_10_Krankheiten_des_Atmungssystems	Numeric	0	0	Missing Values Imputed	Imputed value: 0
Secondary_Diagnoses_icd_10_kapitel_7_Krankheiten_des_Auges_und_der_Augenanhangsgebilde	Numeric	0	0	Missing Values Imputed	Imputed value: 0
Secondary_Diagnoses_icd_10_kapitel_16_Bestimmte_Zustaende_die_ihren_Ursprung_in_der_Perinatalperiode_haben	Numeric	0	0	Missing Values Imputed	Imputed value: 0
Secondary_Diagnoses_icd_10_kapitel_5_Psychische_und_Verhaltensstoerungen	Numeric	0	0	Missing Values Imputed	Imputed value: 0
Secondary_Diagnoses_icd_10_kapitel_null	Numeric	0	0	Missing Values Imputed	Imputed value: 0
Secondary_Diagnoses_icd_10_kapitel_13_Krankheiten_des_Muskel_Skelett_Systems_und_des_Bindegewebes	Numeric	0	0	Missing Values Imputed	Imputed value: 0
Secondary_Diagnoses_icd_10_kapitel_17_Angeborene_Fehlbildungen_Deformitaeten_und_Chromosomenanomalien	Numeric	0	0	Missing Values Imputed	Imputed value: 0

Secondary_Diagnoses_icd_10_kapitel_14_Krankheiten_des_Urogenitalsystems	Numeric	0	0	Missing Values Imputed	Imputed value: 0
Secondary_Diagnoses_icd_10_kapitel_15_Schwangerschaft_Geburt_und_Wochenbett	Numeric	0	0	Missing Values Imputed	Imputed value: 0
Secondary_Diagnoses_icd_10_kapitel_19_Verletzungen_Vergiftungen_und_bestimmte_andere_Folgen_aeusserer_Ursachen	Numeric	0	0	Missing Values Imputed	Imputed value: 0
Secondary_Diagnoses_icd_10_kapitel_21_Faktoren_die_den_Gesundheitszustand_beeinflussen_und_zur_Inanspruchnahme_des_Gesundheitswesens_fuehren	Numeric	0	0	Missing Values Imputed	Imputed value: 0
Secondary_Diagnoses_icd_10_kapitel_20_Äussere_Ursachen_von_Morbiditaet_und_Mortalitaet	Numeric	0	0	Missing Values Imputed	Imputed value: 0
Secondary_Diagnoses_icd_10_kapitel_9_Krankheiten_des_Kreislaufsystems	Numeric	0	0	Missing Values Imputed	Imputed value: 0
Secondary_Diagnoses_icd_10_kapitel_3_Krankheiten_des_Blutes_und_der_blutbildenden_Organe_sowie_bestimmte_Stoerungen_mit_Beteiligung_des_Immunsystems	Numeric	0	0	Missing Values Imputed	Imputed value: 0

eXtreme Gradient Boosted Trees Classifier with Early Stopping (Fast Feature Binning)

Feature Name	Var Type	Missing Count	Missing Percentage	Imputation Name	Imputation Description
Accident_ID	Categorical	359107	99	Ordinal encoding of categorical variables	Imputed value: -2
Education_level_ID	Categorical	4646	1	Ordinal encoding of categorical variables	Imputed value: -2

Admission_type_ID	Categorical	0	0	Ordinal encoding of categorical variables	Imputed value: -2
Citizenship_ID	Categorical	0	0	Ordinal encoding of categorical variables	Imputed value: -2
County__administrative_region__of_patient	Categorical	0	0	Ordinal encoding of categorical variables	Imputed value: -2
NUTS2_region_of_hospital	Categorical	0	0	Ordinal encoding of categorical variables	Imputed value: -2
NUTS2_region_of_patient	Categorical	0	0	Ordinal encoding of categorical variables	Imputed value: -2
Insurance_type_ID	Categorical	0	0	Ordinal encoding of categorical variables	Imputed value: -2
Sex	Categorical	0	0	Ordinal encoding of categorical variables	Imputed value: -2
governmental_measures_randomized_phase_id (Categorical Int)	Categorical	0	0	Ordinal encoding of categorical variables	Imputed value: -2
is_romanian	Numeric	0	0	Missing Values Imputed	Imputed value: 1

has_positive_test	Numeric	0	0	Missing Values Imputed	Imputed value: 0
has_covid_suspected_or_positive_test	Numeric	0	0	Missing Values Imputed	Imputed value: 0
Age	Numeric	0	0	Missing Values Imputed	Imputed value: 53
Secondary_Diagnoses_icd_10_kapitel_4_Endokrine_Ernaehrungs__und_Stoffwechselkrankheiten	Numeric	0	0	Missing Values Imputed	Imputed value: 0
Secondary_Diagnoses_icd_10_kapitel_1_Bestimmte_infektioese_und_parasitaere_Krankheiten	Numeric	0	0	Missing Values Imputed	Imputed value: 0
Secondary_Diagnoses_icd_10_kapitel_12_Krankheiten_der_Haut_und_der_Unterhaut	Numeric	0	0	Missing Values Imputed	Imputed value: 0
Secondary_Diagnoses_icd_10_kapitel_22_Schluesselnummern_fuer_besondere_Zwecke	Numeric	0	0	Missing Values Imputed	Imputed value: 0
Secondary_Diagnoses_icd_10_kapitel_6_Krankheiten_des_Nervensystems	Numeric	0	0	Missing Values Imputed	Imputed value: 0
Secondary_Diagnoses_icd_10_kapitel_8_Krankheiten_des_Ohres_und_des_Warzenfortsatzes	Numeric	0	0	Missing Values Imputed	Imputed value: 0
Secondary_Diagnoses_icd_10_kapitel_2_Neubildungen	Numeric	0	0	Missing Values Imputed	Imputed value: 0
Secondary_Diagnoses_icd_10_kapitel_11_Krankheiten_des_Verdauungssystems	Numeric	0	0	Missing Values Imputed	Imputed value: 0

Secondary_Diagnoses_icd_10_kapitel_18_Symptome_und_abnorme_klinische_und_Laborbefunde_die_anderenorts_nicht_klassifiziert_sind	Numeric	0	0	Missing Values Imputed	Imputed value: 0
Secondary_Diagnoses_icd_10_kapitel_10_Krankheiten_des_Atmungssystems	Numeric	0	0	Missing Values Imputed	Imputed value: 0
Secondary_Diagnoses_icd_10_kapitel_7_Krankheiten_des_Auges_und_der_Augenanhangsgebilde	Numeric	0	0	Missing Values Imputed	Imputed value: 0
Secondary_Diagnoses_icd_10_kapitel_16_Bestimmte_Zustaende_die_ihren_Ursprung_in_der_Perinatalperiode_haben	Numeric	0	0	Missing Values Imputed	Imputed value: 0
Secondary_Diagnoses_icd_10_kapitel_5_Psychische_und_Verhaltensstoerungen	Numeric	0	0	Missing Values Imputed	Imputed value: 0
Secondary_Diagnoses_icd_10_kapitel_null	Numeric	0	0	Missing Values Imputed	Imputed value: 0
Secondary_Diagnoses_icd_10_kapitel_13_Krankheiten_des_Muskel_Skelett_Systems_und_des_Bindegewebes	Numeric	0	0	Missing Values Imputed	Imputed value: 0
Secondary_Diagnoses_icd_10_kapitel_17_Angeborene_Fehlbildungen_Deformitaeten_und_Chromosomenanomalien	Numeric	0	0	Missing Values Imputed	Imputed value: 0
Secondary_Diagnoses_icd_10_kapitel_14_Krankheiten_des_Urogenitalsystems	Numeric	0	0	Missing Values Imputed	Imputed value: 0
Secondary_Diagnoses_icd_10_kapitel_15_Schwangerschaft_Geburt_und_Wochenbett	Numeric	0	0	Missing Values Imputed	Imputed value: 0
Secondary_Diagnoses_icd_10_kapitel_19_Verletzungen_Vergiftungen_und_bestimmte_andere_Folgen_aeusserer_Ursachen	Numeric	0	0	Missing Values Imputed	Imputed value: 0

Secondary_Diagnoses_icd_10_kapitel_21_Faktoren_die_den_Gesundheitszustand_beeinflussen_und_zur_Inanspruchnahme_des_Gesundheitswesens_fuehren	Numeric	0	0	Missing Values Imputed	Imputed value: 0
Secondary_Diagnoses_icd_10_kapitel_20_Äussere_Ursachen_von_Morbiditaet_und_Mortalitaet	Numeric	0	0	Missing Values Imputed	Imputed value: 0
Secondary_Diagnoses_icd_10_kapitel_9_Krankheiten_des_Kreislaufsystems	Numeric	0	0	Missing Values Imputed	Imputed value: 0
Secondary_Diagnoses_icd_10_kapitel_3_Krankheiten_des_Blutes_und_der_blutbildenden_Organe_sowie_bestimmte_Stoerungen_mit_Beteiligung_des_Immunsystems	Numeric	0	0	Missing Values Imputed	Imputed value: 0

eXtreme Gradient Boosted Trees Classifier with Early Stopping (Fast Feature Binning) and Unsupervised Learning Features

Feature Name	Var Type	Missing Count	Missing Percentage	Imputation Name	Imputation Description
Accident_ID	Categorical	359107	99	Ordinal encoding of categorical variables	Imputed value: -2
Accident_ID	Categorical	359107	99	One-Hot Encoding	Missing indicator treated as feature
Education_level_ID	Categorical	4646	1	Ordinal encoding of categorical variables	Imputed value: -2
Education_level_ID	Categorical	4646	1	One-Hot Encoding	Missing indicator

					treated as feature
Admission_type_ID	Categorical	0	0	Ordinal encoding of categorical variables	Imputed value: -2
Admission_type_ID	Categorical	0	0	One-Hot Encoding	Missing values ignored
Citizenship_ID	Categorical	0	0	Ordinal encoding of categorical variables	Imputed value: -2
Citizenship_ID	Categorical	0	0	One-Hot Encoding	Missing values treated as infrequent
County__administrative_region__of_patient	Categorical	0	0	Ordinal encoding of categorical variables	Imputed value: -2
County__administrative_region__of_patient	Categorical	0	0	One-Hot Encoding	Missing values ignored
NUTS2_region_of_hospital	Categorical	0	0	Ordinal encoding of categorical variables	Imputed value: -2
NUTS2_region_of_hospital	Categorical	0	0	One-Hot Encoding	Missing values ignored
NUTS2_region_of_patient	Categorical	0	0	Ordinal encoding of categorical variables	Imputed value: -2
NUTS2_region_of_patient	Categorical	0	0	One-Hot Encoding	Missing values ignored

Insurance_type_ID	Categorical	0	0	Ordinal encoding of categorical variables	Imputed value: -2
Insurance_type_ID	Categorical	0	0	One-Hot Encoding	Missing values ignored
Sex	Categorical	0	0	Ordinal encoding of categorical variables	Imputed value: -2
Sex	Categorical	0	0	One-Hot Encoding	Missing values ignored
governmental_measures_randomized_phase_id (Categorical Int)	Categorical	0	0	Ordinal encoding of categorical variables	Imputed value: -2
governmental_measures_randomized_phase_id (Categorical Int)	Categorical	0	0	One-Hot Encoding	Missing values ignored
is_romanian	Numeric	0	0	Missing Values Imputed	Imputed value: 1
is_romanian	Numeric	0	0	Missing Values Imputed	Imputed value: 1
has_positive_test	Numeric	0	0	Missing Values Imputed	Imputed value: 0
has_positive_test	Numeric	0	0	Missing Values Imputed	Imputed value: 0
has_covid_suspected_or_positive_test	Numeric	0	0	Missing Values Imputed	Imputed value: 0

has_covid_suspected_or_positive_test	Numeric	0	0	Missing Values Imputed	Imputed value: 0
Age	Numeric	0	0	Missing Values Imputed	Imputed value: 53
Age	Numeric	0	0	Missing Values Imputed	Imputed value: 53
Secondary_Diagnoses_icd_10_kapitel_4_Endokrine_Ernaehrungs__und_Stoffwechselkrankheiten	Numeric	0	0	Missing Values Imputed	Imputed value: 0
Secondary_Diagnoses_icd_10_kapitel_4_Endokrine_Ernaehrungs__und_Stoffwechselkrankheiten	Numeric	0	0	Missing Values Imputed	Imputed value: 0
Secondary_Diagnoses_icd_10_kapitel_1_Bestimmte_infektioese_und_parasitaere_Krankheiten	Numeric	0	0	Missing Values Imputed	Imputed value: 0
Secondary_Diagnoses_icd_10_kapitel_1_Bestimmte_infektioese_und_parasitaere_Krankheiten	Numeric	0	0	Missing Values Imputed	Imputed value: 0
Secondary_Diagnoses_icd_10_kapitel_12_Krankheiten_der_Haut_und_der_Unterhaut	Numeric	0	0	Missing Values Imputed	Imputed value: 0
Secondary_Diagnoses_icd_10_kapitel_12_Krankheiten_der_Haut_und_der_Unterhaut	Numeric	0	0	Missing Values Imputed	Imputed value: 0
Secondary_Diagnoses_icd_10_kapitel_22_Schluesselnummern_fuer_besondere_Zwecke	Numeric	0	0	Missing Values Imputed	Imputed value: 0
Secondary_Diagnoses_icd_10_kapitel_22_Schluesselnummern_fuer_besondere_Zwecke	Numeric	0	0	Missing Values Imputed	Imputed value: 0

Secondary_Diagnoses_icd_10_kapitel_6_Krankheiten_des_Nervensystems	Numeric	0	0	Missing Values Imputed	Imputed value: 0
Secondary_Diagnoses_icd_10_kapitel_6_Krankheiten_des_Nervensystems	Numeric	0	0	Missing Values Imputed	Imputed value: 0
Secondary_Diagnoses_icd_10_kapitel_8_Krankheiten_des_Ohres_und_des_Warzenfortsatzes	Numeric	0	0	Missing Values Imputed	Imputed value: 0
Secondary_Diagnoses_icd_10_kapitel_8_Krankheiten_des_Ohres_und_des_Warzenfortsatzes	Numeric	0	0	Missing Values Imputed	Imputed value: 0
Secondary_Diagnoses_icd_10_kapitel_2_Neubildungen	Numeric	0	0	Missing Values Imputed	Imputed value: 0
Secondary_Diagnoses_icd_10_kapitel_2_Neubildungen	Numeric	0	0	Missing Values Imputed	Imputed value: 0
Secondary_Diagnoses_icd_10_kapitel_11_Krankheiten_des_Verdauungssystems	Numeric	0	0	Missing Values Imputed	Imputed value: 0
Secondary_Diagnoses_icd_10_kapitel_11_Krankheiten_des_Verdauungssystems	Numeric	0	0	Missing Values Imputed	Imputed value: 0
Secondary_Diagnoses_icd_10_kapitel_18_Symptome_und_abnorme_klinische_und_Laborbefunde_die_anderenorts_nicht_klassifiziert_sind	Numeric	0	0	Missing Values Imputed	Imputed value: 0
Secondary_Diagnoses_icd_10_kapitel_18_Symptome_und_abnorme_klinische_und_Laborbefunde_die_anderenorts_nicht_klassifiziert_sind	Numeric	0	0	Missing Values Imputed	Imputed value: 0
Secondary_Diagnoses_icd_10_kapitel_10_Krankheiten_des_Atmungssystems	Numeric	0	0	Missing Values Imputed	Imputed value: 0

Secondary_Diagnoses_icd_10_kapitel_10_Krankheiten_des_Atmungssystems	Numeric	0	0	Missing Values Imputed	Imputed value: 0
Secondary_Diagnoses_icd_10_kapitel_7_Krankheiten_des_Auges_und_der_Augenanhangsgebilde	Numeric	0	0	Missing Values Imputed	Imputed value: 0
Secondary_Diagnoses_icd_10_kapitel_7_Krankheiten_des_Auges_und_der_Augenanhangsgebilde	Numeric	0	0	Missing Values Imputed	Imputed value: 0
Secondary_Diagnoses_icd_10_kapitel_16_Bestimmte_Zustaende_die_ihren_Ursprung_in_der_Perinatalperiode_haben	Numeric	0	0	Missing Values Imputed	Imputed value: 0
Secondary_Diagnoses_icd_10_kapitel_16_Bestimmte_Zustaende_die_ihren_Ursprung_in_der_Perinatalperiode_haben	Numeric	0	0	Missing Values Imputed	Imputed value: 0
Secondary_Diagnoses_icd_10_kapitel_5_Psychische_und_Verhaltensstoerungen	Numeric	0	0	Missing Values Imputed	Imputed value: 0
Secondary_Diagnoses_icd_10_kapitel_5_Psychische_und_Verhaltensstoerungen	Numeric	0	0	Missing Values Imputed	Imputed value: 0
Secondary_Diagnoses_icd_10_kapitel_null	Numeric	0	0	Missing Values Imputed	Imputed value: 0
Secondary_Diagnoses_icd_10_kapitel_null	Numeric	0	0	Missing Values Imputed	Imputed value: 0
Secondary_Diagnoses_icd_10_kapitel_13_Krankheiten_des_Muskel_Skelett_Systems_und_des_Bindegewebes	Numeric	0	0	Missing Values Imputed	Imputed value: 0
Secondary_Diagnoses_icd_10_kapitel_13_Krankheiten_des_Muskel_Skelett_Systems_und_des_Bindegewebes	Numeric	0	0	Missing Values Imputed	Imputed value: 0

Secondary_Diagnoses_icd_10_kapitel_17_Angeborene_Fehlbildungen_Deformitaeten_und_Chromosomenanomalien	Numeric	0	0	Missing Values Imputed	Imputed value: 0
Secondary_Diagnoses_icd_10_kapitel_17_Angeborene_Fehlbildungen_Deformitaeten_und_Chromosomenanomalien	Numeric	0	0	Missing Values Imputed	Imputed value: 0
Secondary_Diagnoses_icd_10_kapitel_14_Krankheiten_des_Urogenitalsystems	Numeric	0	0	Missing Values Imputed	Imputed value: 0
Secondary_Diagnoses_icd_10_kapitel_14_Krankheiten_des_Urogenitalsystems	Numeric	0	0	Missing Values Imputed	Imputed value: 0
Secondary_Diagnoses_icd_10_kapitel_15_Schwangerschaft_Geburt_und_Wochenbett	Numeric	0	0	Missing Values Imputed	Imputed value: 0
Secondary_Diagnoses_icd_10_kapitel_15_Schwangerschaft_Geburt_und_Wochenbett	Numeric	0	0	Missing Values Imputed	Imputed value: 0
Secondary_Diagnoses_icd_10_kapitel_19_Verletzungen_Vergiftungen_und_bestimmte_andere_Folgen_aeusserer_Ursachen	Numeric	0	0	Missing Values Imputed	Imputed value: 0
Secondary_Diagnoses_icd_10_kapitel_19_Verletzungen_Vergiftungen_und_bestimmte_andere_Folgen_aeusserer_Ursachen	Numeric	0	0	Missing Values Imputed	Imputed value: 0
Secondary_Diagnoses_icd_10_kapitel_21_Faktoren_die_den_Gesundheitszustand_beeinflussen_und_zur_Inanspruchnahme_des_Gesundheitswesens_fuehren	Numeric	0	0	Missing Values Imputed	Imputed value: 0
Secondary_Diagnoses_icd_10_kapitel_21_Faktoren_die_den_Gesundheitszustand_beeinflussen_und_zur_Inanspruchnahme_des_Gesundheitswesens_fuehren	Numeric	0	0	Missing Values Imputed	Imputed value: 0
Secondary_Diagnoses_icd_10_kapitel_20_Äussere_Ursachen_von_Morbiditaet_und_Mortalitaet	Numeric	0	0	Missing Values Imputed	Imputed value: 0

Secondary_Diagnoses_icd_10_kapitel_20_Äussere_Ursachen_von_Morbiditaet_und_Mortalitaet	Numeric	0	0	Missing Values Imputed	Imputed value: 0
Secondary_Diagnoses_icd_10_kapitel_9_Krankheiten_des_Kreislaufsystems	Numeric	0	0	Missing Values Imputed	Imputed value: 0
Secondary_Diagnoses_icd_10_kapitel_9_Krankheiten_des_Kreislaufsystems	Numeric	0	0	Missing Values Imputed	Imputed value: 0
Secondary_Diagnoses_icd_10_kapitel_3_Krankheiten_des_Blutes_und_der_blutbildenden_Organe_sowie_bestimmte_Stoerungen_mit_Beteiligung_des_Immunsystems	Numeric	0	0	Missing Values Imputed	Imputed value: 0
Secondary_Diagnoses_icd_10_kapitel_3_Krankheiten_des_Blutes_und_der_blutbildenden_Organe_sowie_bestimmte_Stoerungen_mit_Beteiligung_des_Immunsystems	Numeric	0	0	Missing Values Imputed	Imputed value: 0

Target 4: has COVID-19

Features for Modeling and Summary Statistics

Feature Name	Var Type	Unique	Missing	Mean	Std Dev	Median	Min	Max	Target Leakage
Main_Procedure	Categorical	2340	203604	N/A	N/A	N/A	N/A	N/A	Low
Admission_type_ID	Categorical	6	0	N/A	N/A	N/A	N/A	N/A	Low
Hospital_ID	Categorical	504	0	N/A	N/A	N/A	N/A	N/A	Low
Admission_Date	Date	120109	0	2020-07-22T21:27:18.960787	37.54 days	2020-07-22T18:27:00	2020-05-15T00:00:00	2020-09-30T15:31:00	Low

Date_of_main_procedure	Date	36944	203610	2020-07-23T10:07:24.120500	37.66 days	2020-07-22T08:55:37	2020-05-15T00:20:00	2020-09-30T15:30:00	Low
Acute_case_or_emergency	Boolean	2	0	0.93	0.26	1.0	0.0	1.0	Low
Medical_Specialist_ID	Categorical	46	3338	N/A	N/A	N/A	N/A	N/A	Low
County__administrative_region__of_patient	Categorical	42	0	N/A	N/A	N/A	N/A	N/A	Low
State_at_discharge_ID	Categorical	5	0	N/A	N/A	N/A	N/A	N/A	Low
Occupation_ID	Categorical	8	25202	N/A	N/A	N/A	N/A	N/A	Low
Admission_ward_ID	Categorical	97	0	N/A	N/A	N/A	N/A	N/A	Low
NUTS2_region_of_hospital	Categorical	8	0	N/A	N/A	N/A	N/A	N/A	Low
NUTS2_region_of_patient	Categorical	8	0	N/A	N/A	N/A	N/A	N/A	Low
Admission_criteria_ID	Categorical	6	2	N/A	N/A	N/A	N/A	N/A	Low
Main_Diagnosis__second_	Categorical	815	261046	N/A	N/A	N/A	N/A	N/A	Low
is_cured_or_ameliorated	Boolean	2	0	0.87	0.34	1.0	0.0	1.0	Low
Age	Numeric	104	0	47.29	25.56	52.0	-7.0	102.0	Low
governmental_measures_randomized_phase_id	Numeric	10	0	43.56	30.28	37.0	3.0	100.0	Low
Education_level_ID	Categorical	9	3664	N/A	N/A	N/A	N/A	N/A	Low
Secondary_Diagnoses_icd_10_kapitel_4_Endokrine_Ernaehrungs__und_Stoffwechselkrankheiten	Boolean	2	0	0.32	0.47	0.0	0.0	1.0	Low
Secondary_Diagnoses_icd_10_kapitel_1_Bestimmte_infektioese_und_parasitaere_Krankheiten	Boolean	2	0	0.15	0.35	0.0	0.0	1.0	Low
Secondary_Diagnoses_icd_10_kapitel_12_Krankheiten_der_Haut_und_der_Unterhaut	Boolean	2	0	0.032	0.18	0.0	0.0	1.0	Low
Secondary_Diagnoses_icd_10_kapitel_6_Krankheiten_des_Nervensystems	Boolean	2	0	0.065	0.25	0.0	0.0	1.0	Low

Secondary_Diagnoses_icd_10_kapitel_8_Krankheiten_des_Ohres_und_des_Warzenfortsatzes	Boolean	2	0	0.015	0.12	0.0	0.0	1.0	Low
Secondary_Diagnoses_icd_10_kapitel_2_Neubildungen	Boolean	2	0	0.099	0.3	0.0	0.0	1.0	Low
Secondary_Diagnoses_icd_10_kapitel_11_Krankheiten_des_Verdauungssystems	Boolean	2	0	0.18	0.38	0.0	0.0	1.0	Low
Secondary_Diagnoses_icd_10_kapitel_18_Symptome_und_abnorme_klinische_und_Laborbefunde_die_anderenorts_nicht_klassifiziert_sind	Boolean	2	0	0.25	0.43	0.0	0.0	1.0	Low
Secondary_Diagnoses_icd_10_kapitel_10_Krankheiten_des_Atmungssystems	Boolean	2	0	0.16	0.36	0.0	0.0	1.0	Low
Secondary_Diagnoses_icd_10_kapitel_7_Krankheiten_des_Auges_und_der_Augenanhangsgebilde	Boolean	2	0	0.0205	0.14	0.0	0.0	1.0	Low
Secondary_Diagnoses_icd_10_kapitel_16_Bestimmte_Zustaende_die_ihren_Ursprung_in_der_Perinatalperiode_haben	Boolean	2	0	0.066	0.25	0.0	0.0	1.0	Low
Secondary_Diagnoses_icd_10_kapitel_5_Psychische_und_Verhaltensstoerungen	Boolean	2	0	0.107	0.31	0.0	0.0	1.0	Low
Secondary_Diagnoses_icd_10_kapitel_null	Boolean	1	0	1.0	0.0	1.0	1.0	1.0	N/A
Secondary_Diagnoses_icd_10_kapitel_13_Krankheiten_des_Muskel_Skelett_Systems_und_des_Bindegewebes	Boolean	2	0	0.093	0.29	0.0	0.0	1.0	Low
Secondary_Diagnoses_icd_10_kapitel_17_Angeborene_Fehlbildungen_Deformitaeten_und_Chromosomenanomalien	Boolean	2	0	0.027	0.16	0.0	0.0	1.0	Low
Secondary_Diagnoses_icd_10_kapitel_14_Krankheiten_des_Urogenitalsystems	Boolean	2	0	0.13	0.34	0.0	0.0	1.0	Low
Secondary_Diagnoses_icd_10_kapitel_15_Schwangerschaft_Geburt_und_Wochenbett	Boolean	2	0	0.09	0.29	0.0	0.0	1.0	Low
Secondary_Diagnoses_icd_10_kapitel_19_Verletzungen_Vergiftungen_und_bestimmte_andere_Folgen_aeusserer_Ursachen	Boolean	2	0	0.058	0.23	0.0	0.0	1.0	Low

Secondary_Diagnoses_icd_10_kapitel_21_Faktoren_die_den_Gesundheitszustand_beeinflussen_und_zur_Inanspruchnahme_des_Gesundheitswesens_fuehren	Boolean	2	0	0.37	0.48	0.0	0.0	1.0	Low
Secondary_Diagnoses_icd_10_kapitel_20_Äussere_Ursachen_von_Morbiditaet_und_Mortalitaet	Boolean	2	0	0.0002	0.016	0.0	0.0	1.0	N/A
Secondary_Diagnoses_icd_10_kapitel_9_Krankheiten_des_Kreislaufsystems	Boolean	2	0	0.37	0.48	0.0	0.0	1.0	Low
Secondary_Diagnoses_icd_10_kapitel_3_Krankheiten_des_Blutes_und_der_blutbildenden_Organe_sowie_bestimmte_Stoerungen_mit_Beteiligung_des_Immunsystems	Boolean	2	0	0.16	0.37	0.0	0.0	1.0	Low

Data Quality Handling Report

Light Gradient Boosted Trees Classifier with Early Stopping

Feature Name	Var Type	Missing Count	Missing Percentage	Imputation Name	Imputation Description
Main_Diagnosis__second_	Categorical	307206	94	Ordinal encoding of categorical variables	Imputed value: -2
Date_of_main_procedure (Day of Week)	Categorical	239673	73	Ordinal encoding of categorical variables	Imputed value: -2
Date_of_main_procedure (Hour of Day)	Categorical	239673	73	Ordinal encoding of categorical variables	Imputed value: -2
Date_of_main_procedure	Numeric	239673	73	Missing Values Imputed	Imputed value: -9999
Main_Procedure	Categorical	239666	73	Ordinal encoding of	Imputed value: -2

				categorical variables	
Occupation_ID	Categorical	29643	9	Ordinal encoding of categorical variables	Imputed value: -2
Education_level_ID	Categorical	4252	1	Ordinal encoding of categorical variables	Imputed value: -2
Medical_Specialist_ID	Categorical	3970	1	Ordinal encoding of categorical variables	Imputed value: -2
Admission_criteria_ID	Categorical	2	0	Ordinal encoding of categorical variables	Imputed value: -2
Admission_type_ID	Categorical	0	0	Ordinal encoding of categorical variables	Imputed value: -2
Hospital_ID	Categorical	0	0	Ordinal encoding of categorical variables	Imputed value: -2
County__administrative_region__of_patient	Categorical	0	0	Ordinal encoding of categorical variables	Imputed value: -2
State_at_discharge_ID	Categorical	0	0	Ordinal encoding of categorical variables	Imputed value: -2
Admission_ward_ID	Categorical	0	0	Ordinal encoding of	Imputed value: -2

				categorical variables	
NUTS2_region_of_hospital	Categorical	0	0	Ordinal encoding of categorical variables	Imputed value: -2
NUTS2_region_of_patient	Categorical	0	0	Ordinal encoding of categorical variables	Imputed value: -2
Admission_Date (Day of Week)	Categorical	0	0	Ordinal encoding of categorical variables	Imputed value: -2
Admission_Date (Hour of Day)	Categorical	0	0	Ordinal encoding of categorical variables	Imputed value: -2
governmental_measures_randomized_phase_id (Categorical Int)	Categorical	0	0	Ordinal encoding of categorical variables	Imputed value: -2
Admission_Date	Numeric	0	0	Missing Values Imputed	Imputed value: 1.5954567e+12
Acute_case_or_emergency	Numeric	0	0	Missing Values Imputed	Imputed value: 1
is_cured_or_ameliorated	Numeric	0	0	Missing Values Imputed	Imputed value: 1
Age	Numeric	0	0	Missing Values Imputed	Imputed value: 52
Secondary_Diagnoses_icd_10_kapitel_4_Endokrine_Ernaehrungs__und_Stoffwechselkrankheiten	Numeric	0	0	Missing Values Imputed	Imputed value: 0
Secondary_Diagnoses_icd_10_kapitel_1_Bestimmte_infektioese_und_parasitaere_Krankheiten	Numeric	0	0	Missing Values Imputed	Imputed value: 0

Secondary_Diagnoses_icd_10_kapitel_12_Krankheiten_der_Haut_und_der_Unterhaut	Numeric	0	0	Missing Values Imputed	Imputed value: 0
Secondary_Diagnoses_icd_10_kapitel_6_Krankheiten_des_Nervensystems	Numeric	0	0	Missing Values Imputed	Imputed value: 0
Secondary_Diagnoses_icd_10_kapitel_8_Krankheiten_des_Ohres_und_des_Warzenfortsatzes	Numeric	0	0	Missing Values Imputed	Imputed value: 0
Secondary_Diagnoses_icd_10_kapitel_2_Neubildungen	Numeric	0	0	Missing Values Imputed	Imputed value: 0
Secondary_Diagnoses_icd_10_kapitel_11_Krankheiten_des_Verdauungssystems	Numeric	0	0	Missing Values Imputed	Imputed value: 0
Secondary_Diagnoses_icd_10_kapitel_18_Symptome_und_abnorme_klinische_und_Laborbefunde_die_anderenorts_nicht_klassifiziert_sind	Numeric	0	0	Missing Values Imputed	Imputed value: 0
Secondary_Diagnoses_icd_10_kapitel_10_Krankheiten_des_Atmungssystems	Numeric	0	0	Missing Values Imputed	Imputed value: 0
Secondary_Diagnoses_icd_10_kapitel_7_Krankheiten_des_Auges_und_der_Augenanhangsgebilde	Numeric	0	0	Missing Values Imputed	Imputed value: 0
Secondary_Diagnoses_icd_10_kapitel_16_Bestimmte_Zustaende_die_ihren_Ursprung_in_der_Perinatalperiode_haben	Numeric	0	0	Missing Values Imputed	Imputed value: 0
Secondary_Diagnoses_icd_10_kapitel_5_Psychische_und_Verhaltensstoerungen	Numeric	0	0	Missing Values Imputed	Imputed value: 0
Secondary_Diagnoses_icd_10_kapitel_null	Numeric	0	0	Missing Values Imputed	Imputed value: 0
Secondary_Diagnoses_icd_10_kapitel_13_Krankheiten_des_Muskel_Skelett_Systems_und_des_Bindegewebes	Numeric	0	0	Missing Values Imputed	Imputed value: 0
Secondary_Diagnoses_icd_10_kapitel_17_Angeborene_Fehlbildungen_Deformitaeten_und_Chromosomenanomalien	Numeric	0	0	Missing Values Imputed	Imputed value: 0
Secondary_Diagnoses_icd_10_kapitel_14_Krankheiten_des_Urogenitalsystems	Numeric	0	0	Missing Values Imputed	Imputed value: 0
Secondary_Diagnoses_icd_10_kapitel_15_Schwangerschaft_Geburt_und_Wochenbett	Numeric	0	0	Missing Values Imputed	Imputed value: 0

Secondary_Diagnoses_icd_10_kapitel_19_Verletzungen_Vergiftungen_und_bestimmte_andere_Folgen_aeusserer_Ursachen	Numeric	0	0	Missing Values Imputed	Imputed value: 0
Secondary_Diagnoses_icd_10_kapitel_21_Faktoren_die_den_Gesundheitszustand_beeinflussen_und_zur_Inanspruchnahme_des_Gesundheitswesens_fuehren	Numeric	0	0	Missing Values Imputed	Imputed value: 0
Secondary_Diagnoses_icd_10_kapitel_20_Äussere_Ursachen_von_Morbiditaet_und_Mortalitaet	Numeric	0	0	Missing Values Imputed	Imputed value: 0
Secondary_Diagnoses_icd_10_kapitel_9_Krankheiten_des_Kreislaufsystems	Numeric	0	0	Missing Values Imputed	Imputed value: 0
Secondary_Diagnoses_icd_10_kapitel_3_Krankheiten_des_Blutes_und_der_blutbildenden_Organe_sowie_bestimmte_Stoerungen_mit_Beteiligung_des_Immunsystems	Numeric	0	0	Missing Values Imputed	Imputed value: 0

Light Gradient Boosting on ElasticNet Predictions

Feature Name	Var Type	Missing Count	Missing Percentage	Imputation Name	Imputation Description
Main_Diagnosis__second_	Categorical	307206	94	One-Hot Encoding	Missing indicator treated as feature
Main_Diagnosis__second_	Categorical	307206	94	Ordinal encoding of categorical variables	Imputed value: -2
Date_of_main_procedure (Day of Week)	Categorical	239673	73	One-Hot Encoding	Missing indicator treated as feature
Date_of_main_procedure (Day of Week)	Categorical	239673	73	Ordinal encoding of categorical variables	Imputed value: -2

Date_of_main_procedure (Hour of Day)	Categorical	239673	73	One-Hot Encoding	Missing indicator treated as feature
Date_of_main_procedure (Hour of Day)	Categorical	239673	73	Ordinal encoding of categorical variables	Imputed value: -2
Date_of_main_procedure	Numeric	239673	73	Missing Values Imputed	Missing indicator treated as feature, Imputed value: 1.5954105e+12
Main_Procedure	Categorical	239666	73	One-Hot Encoding	Missing indicator treated as feature
Main_Procedure	Categorical	239666	73	Ordinal encoding of categorical variables	Imputed value: -2
Occupation_ID	Categorical	29643	9	One-Hot Encoding	Missing indicator treated as feature
Occupation_ID	Categorical	29643	9	Ordinal encoding of categorical variables	Imputed value: -2
Education_level_ID	Categorical	4252	1	One-Hot Encoding	Missing indicator treated as feature

Education_level_ID	Categorical	4252	1	Ordinal encoding of categorical variables	Imputed value: -2
Medical_Specialist_ID	Categorical	3970	1	One-Hot Encoding	Missing indicator treated as feature
Medical_Specialist_ID	Categorical	3970	1	Ordinal encoding of categorical variables	Imputed value: -2
Admission_criteria_ID	Categorical	2	0	One-Hot Encoding	Missing values treated as infrequent
Admission_criteria_ID	Categorical	2	0	Ordinal encoding of categorical variables	Imputed value: -2
Admission_type_ID	Categorical	0	0	One-Hot Encoding	Missing values ignored
Admission_type_ID	Categorical	0	0	Ordinal encoding of categorical variables	Imputed value: -2
Hospital_ID	Categorical	0	0	One-Hot Encoding	Missing values treated as infrequent
Hospital_ID	Categorical	0	0	Ordinal encoding of categorical variables	Imputed value: -2

County__administrative_region__of_patient	Categorical	0	0	One-Hot Encoding	Missing values ignored
County__administrative_region__of_patient	Categorical	0	0	Ordinal encoding of categorical variables	Imputed value: -2
State_at_discharge_ID	Categorical	0	0	One-Hot Encoding	Missing values ignored
State_at_discharge_ID	Categorical	0	0	Ordinal encoding of categorical variables	Imputed value: -2
Admission_ward_ID	Categorical	0	0	One-Hot Encoding	Missing values treated as infrequent
Admission_ward_ID	Categorical	0	0	Ordinal encoding of categorical variables	Imputed value: -2
NUTS2_region_of_hospital	Categorical	0	0	One-Hot Encoding	Missing values ignored
NUTS2_region_of_hospital	Categorical	0	0	Ordinal encoding of categorical variables	Imputed value: -2
NUTS2_region_of_patient	Categorical	0	0	One-Hot Encoding	Missing values ignored
NUTS2_region_of_patient	Categorical	0	0	Ordinal encoding of	Imputed value: -2

				categorical variables	
Admission_Date (Day of Week)	Categorical	0	0	One-Hot Encoding	Missing values ignored
Admission_Date (Day of Week)	Categorical	0	0	Ordinal encoding of categorical variables	Imputed value: -2
Admission_Date (Hour of Day)	Categorical	0	0	One-Hot Encoding	Missing values ignored
Admission_Date (Hour of Day)	Categorical	0	0	Ordinal encoding of categorical variables	Imputed value: -2
governmental_measures_randomized_phase_id (Categorical Int)	Categorical	0	0	One-Hot Encoding	Missing values ignored
governmental_measures_randomized_phase_id (Categorical Int)	Categorical	0	0	Ordinal encoding of categorical variables	Imputed value: -2
Admission_Date	Numeric	0	0	Missing Values Imputed	Imputed value: 1.5954567e+12
Acute_case_or_emergency	Numeric	0	0	Missing Values Imputed	Imputed value: 1
is_cured_or_ameliorated	Numeric	0	0	Missing Values Imputed	Imputed value: 1

Age	Numeric	0	0	Missing Values Imputed	Imputed value: 52
Secondary_Diagnoses_icd_10_kapitel_4_Endokrine_Ernaehrungs__und_Stoffwechselkrankheiten	Numeric	0	0	Missing Values Imputed	Imputed value: 0
Secondary_Diagnoses_icd_10_kapitel_1_Bestimmte_infektioese_und_parasitaere_Krankheiten	Numeric	0	0	Missing Values Imputed	Imputed value: 0
Secondary_Diagnoses_icd_10_kapitel_12_Krankheiten_der_Haut_und_der_Unterhaut	Numeric	0	0	Missing Values Imputed	Imputed value: 0
Secondary_Diagnoses_icd_10_kapitel_6_Krankheiten_des_Nervensystems	Numeric	0	0	Missing Values Imputed	Imputed value: 0
Secondary_Diagnoses_icd_10_kapitel_8_Krankheiten_des_Ohres_und_des_Warzenfortsatzes	Numeric	0	0	Missing Values Imputed	Imputed value: 0
Secondary_Diagnoses_icd_10_kapitel_2_Neubildungen	Numeric	0	0	Missing Values Imputed	Imputed value: 0
Secondary_Diagnoses_icd_10_kapitel_11_Krankheiten_des_Verdauungssystems	Numeric	0	0	Missing Values Imputed	Imputed value: 0
Secondary_Diagnoses_icd_10_kapitel_18_Symptome_und_abnorme_klinische_und_Laborbefunde_die_anderenorts_nicht_klassifiziert_sind	Numeric	0	0	Missing Values Imputed	Imputed value: 0
Secondary_Diagnoses_icd_10_kapitel_10_Krankheiten_des_Atmungssystems	Numeric	0	0	Missing Values Imputed	Imputed value: 0
Secondary_Diagnoses_icd_10_kapitel_7_Krankheiten_des_Auges_und_der_Augenanhangsgebilde	Numeric	0	0	Missing Values Imputed	Imputed value: 0

Secondary_Diagnoses_icd_10_kapitel_16_Bestimmte_Zustaende_die_ihren_Ursprung_in_der_Perinatalperiode_haben	Numeric	0	0	Missing Values Imputed	Imputed value: 0
Secondary_Diagnoses_icd_10_kapitel_5_Psychische_und_Verhaltensstoerungen	Numeric	0	0	Missing Values Imputed	Imputed value: 0
Secondary_Diagnoses_icd_10_kapitel_null	Numeric	0	0	Missing Values Imputed	Imputed value: 0
Secondary_Diagnoses_icd_10_kapitel_13_Krankheiten_des_Muskel_Skelett_Systems_und_des_Bindegewebes	Numeric	0	0	Missing Values Imputed	Imputed value: 0
Secondary_Diagnoses_icd_10_kapitel_17_Angeborene_Fehlbildungen_Deformitaeten_und_Chromosomenanomalien	Numeric	0	0	Missing Values Imputed	Imputed value: 0
Secondary_Diagnoses_icd_10_kapitel_14_Krankheiten_des_Urogenitalsystems	Numeric	0	0	Missing Values Imputed	Imputed value: 0
Secondary_Diagnoses_icd_10_kapitel_15_Schwangerschaft_Geburt_und_Wochenbett	Numeric	0	0	Missing Values Imputed	Imputed value: 0
Secondary_Diagnoses_icd_10_kapitel_19_Verletzungen_Vergiftungen_und_bestimmte_andere_Folgen_aeusserer_Ursachen	Numeric	0	0	Missing Values Imputed	Imputed value: 0
Secondary_Diagnoses_icd_10_kapitel_21_Faktoren_die_den_Gesundheitszustand_beeinflussen_und_zur_Inanspruchnahme_des_Gesundheitswesens_fuehren	Numeric	0	0	Missing Values Imputed	Imputed value: 0
Secondary_Diagnoses_icd_10_kapitel_20_Äussere_Ursachen_von_Morbiditaet_und_Mortalitaet	Numeric	0	0	Missing Values Imputed	Imputed value: 0
Secondary_Diagnoses_icd_10_kapitel_9_Krankheiten_des_Kreislaufsystems	Numeric	0	0	Missing Values Imputed	Imputed value: 0

Secondary_Diagnoses_icd_10_kapitel_3_Krankheiten_des_Blutes_und_der_blutbildenden_Organe_sowie_bestimmte_Stoerungen_mit_Beteiligung_des_Immunsystems	Numeric	0	0	Missing Values Imputed	Imputed value: 0

eXtreme Gradient Boosted Trees Classifier with Early Stopping (Fast Feature Binning)

Feature Name	Var Type	Missing Count	Missing Percentage	Imputation Name	Imputation Description
Main_Diagnosis__second_	Categorical	307206	94	Ordinal encoding of categorical variables	Imputed value: -2
Date_of_main_procedure (Day of Week)	Categorical	239673	73	Ordinal encoding of categorical variables	Imputed value: -2
Date_of_main_procedure (Hour of Day)	Categorical	239673	73	Ordinal encoding of categorical variables	Imputed value: -2
Date_of_main_procedure	Numeric	239673	73	Missing Values Imputed	Imputed value: -9999
Main_Procedure	Categorical	239666	73	Ordinal encoding of categorical variables	Imputed value: -2
Occupation_ID	Categorical	29643	9	Ordinal encoding of categorical variables	Imputed value: -2

Education_level_ID	Categorical	4252	1	Ordinal encoding of categorical variables	Imputed value: -2
Medical_Specialist_ID	Categorical	3970	1	Ordinal encoding of categorical variables	Imputed value: -2
Admission_criteria_ID	Categorical	2	0	Ordinal encoding of categorical variables	Imputed value: -2
Admission_type_ID	Categorical	0	0	Ordinal encoding of categorical variables	Imputed value: -2
Hospital_ID	Categorical	0	0	Ordinal encoding of categorical variables	Imputed value: -2
County__administrative_region__of_patient	Categorical	0	0	Ordinal encoding of categorical variables	Imputed value: -2
State_at_discharge_ID	Categorical	0	0	Ordinal encoding of categorical variables	Imputed value: -2
Admission_ward_ID	Categorical	0	0	Ordinal encoding of	Imputed value: -2

				categorical variables	
NUTS2_region_of_hospital	Categorical	0	0	Ordinal encoding of categorical variables	Imputed value: -2
NUTS2_region_of_patient	Categorical	0	0	Ordinal encoding of categorical variables	Imputed value: -2
Admission_Date (Day of Week)	Categorical	0	0	Ordinal encoding of categorical variables	Imputed value: -2
Admission_Date (Hour of Day)	Categorical	0	0	Ordinal encoding of categorical variables	Imputed value: -2
governmental_measures_randomized_phase_id (Categorical Int)	Categorical	0	0	Ordinal encoding of categorical variables	Imputed value: -2
Admission_Date	Numeric	0	0	Missing Values Imputed	Imputed value: 1.5954567e+12
Acute_case_or_emergency	Numeric	0	0	Missing Values Imputed	Imputed value: 1
is_cured_or_ameliorated	Numeric	0	0	Missing Values Imputed	Imputed value: 1

Age	Numeric	0	0	Missing Values Imputed	Imputed value: 52
Secondary_Diagnoses_icd_10_kapitel_4_Endokrine_Ernaehrungs__und_Stoffwechselkrankheiten	Numeric	0	0	Missing Values Imputed	Imputed value: 0
Secondary_Diagnoses_icd_10_kapitel_1_Bestimmte_infektioese_und_parasitaere_Krankheiten	Numeric	0	0	Missing Values Imputed	Imputed value: 0
Secondary_Diagnoses_icd_10_kapitel_12_Krankheiten_der_Haut_und_der_Unterhaut	Numeric	0	0	Missing Values Imputed	Imputed value: 0
Secondary_Diagnoses_icd_10_kapitel_6_Krankheiten_des_Nervensystems	Numeric	0	0	Missing Values Imputed	Imputed value: 0
Secondary_Diagnoses_icd_10_kapitel_8_Krankheiten_des_Ohres_und_des_Warzenfortsatzes	Numeric	0	0	Missing Values Imputed	Imputed value: 0
Secondary_Diagnoses_icd_10_kapitel_2_Neubildungen	Numeric	0	0	Missing Values Imputed	Imputed value: 0
Secondary_Diagnoses_icd_10_kapitel_11_Krankheiten_des_Verdauungssystems	Numeric	0	0	Missing Values Imputed	Imputed value: 0
Secondary_Diagnoses_icd_10_kapitel_18_Symptome_und_abnorme_klinische_und_Laborbefunde_die_anderenorts_nicht_klassifiziert_sind	Numeric	0	0	Missing Values Imputed	Imputed value: 0
Secondary_Diagnoses_icd_10_kapitel_10_Krankheiten_des_Atmungssystems	Numeric	0	0	Missing Values Imputed	Imputed value: 0
Secondary_Diagnoses_icd_10_kapitel_7_Krankheiten_des_Auges_und_der_Augenanhangsgebilde	Numeric	0	0	Missing Values Imputed	Imputed value: 0

Secondary_Diagnoses_icd_10_kapitel_16_Bestimmte_Zustaende_die_ihren_Ursprung_in_der_Perinatalperiode_haben	Numeric	0	0	Missing Values Imputed	Imputed value: 0
Secondary_Diagnoses_icd_10_kapitel_5_Psychische_und_Verhaltensstoerungen	Numeric	0	0	Missing Values Imputed	Imputed value: 0
Secondary_Diagnoses_icd_10_kapitel_null	Numeric	0	0	Missing Values Imputed	Imputed value: 0
Secondary_Diagnoses_icd_10_kapitel_13_Krankheiten_des_Muskel_Skelett_Systems_und_des_Bindegewebes	Numeric	0	0	Missing Values Imputed	Imputed value: 0
Secondary_Diagnoses_icd_10_kapitel_17_Angeborene_Fehlbildungen_Deformitaeten_und_Chromosomenanomalien	Numeric	0	0	Missing Values Imputed	Imputed value: 0
Secondary_Diagnoses_icd_10_kapitel_14_Krankheiten_des_Urogenitalsystems	Numeric	0	0	Missing Values Imputed	Imputed value: 0
Secondary_Diagnoses_icd_10_kapitel_15_Schwangerschaft_Geburt_und_Wochenbett	Numeric	0	0	Missing Values Imputed	Imputed value: 0
Secondary_Diagnoses_icd_10_kapitel_19_Verletzungen_Vergiftungen_und_bestimmte_andere_Folgen_aeusserer_Ursachen	Numeric	0	0	Missing Values Imputed	Imputed value: 0
Secondary_Diagnoses_icd_10_kapitel_21_Faktoren_die_den_Gesundheitszustand_beeinflussen_und_zur_Inanspruchnahme_des_Gesundheitswesens_fuehren	Numeric	0	0	Missing Values Imputed	Imputed value: 0
Secondary_Diagnoses_icd_10_kapitel_20_Äussere_Ursachen_von_Morbiditaet_und_Mortalitaet	Numeric	0	0	Missing Values Imputed	Imputed value: 0
Secondary_Diagnoses_icd_10_kapitel_9_Krankheiten_des_Kreislaufsystems	Numeric	0	0	Missing Values Imputed	Imputed value: 0

Secondary_Diagnoses_icd_10_kapitel_3_Krankheiten_des_Blutes_und_der_blutbildenden_Organe_sowie_bestimmte_Stoerungen_mit_Beteiligung_des_Immunsystems	Numeric	0	0	Missing Values Imputed	Imputed value: 0

Table S4. Reduced feature lists for each target

**Target "cured or ameliorated"**	
had_intensive_care	Acute_case_or_emergency
Sex	is_romanian
Admission_ward_ID	Citizenship_ID
Accident_ID	Secondary_Diagnoses_icd_10_chapter_12
Admission_type_ID	Secondary_Diagnoses_icd_10_chapter_4
Ventilation_hours	Secondary_Diagnoses_icd_10_chapter_19
Age	Secondary_Diagnoses_icd_10_chapter_11
Governmental_measures_randomized_phase_ID	Secondary_Diagnoses_icd_10_chapter_20
NUTS2_region_of_patient	Secondary_Diagnoses_icd_10_chapter_16
Date_of_main_procedure	Secondary_Diagnoses_icd_10_chapter_2
Date_of_main_procedure_day_of_week	Secondary_Diagnoses_icd_10_chapter_0
Date_of_main_procedure_hour_of_day	Secondary_Diagnoses_icd_10_chapter_8
Discharge_ward_ID	Secondary_Diagnoses_icd_10_chapter_7
has_COVID-19	Secondary_Diagnoses_icd_10_chapter_9
Secondary_Procedure	Secondary_Diagnoses_icd_10_chapter_13
has_COVID-19_suspected_or_performed_test	Secondary_Diagnoses_icd_10_chapter_22
Medical_Specialist_ID	Secondary_Diagnoses_icd_10_chapter_18
Days_spent_in_the_intensive_care_unit	Secondary_Diagnoses_icd_10_chapter_5
had_ventilation	Secondary_Diagnoses_icd_10_chapter_1
County_administrative_region_of_patient	Secondary_Diagnoses_icd_10_chapter_6
Suspected_Diagnosis	Secondary_Diagnoses_icd_10_chapter_21

Education_level_ID	Secondary_Diagnoses_icd_10_chapter_15
has_positive_test	Secondary_Diagnoses_icd_10_chapter_14
Hospital_ID	Secondary_Diagnoses_icd_10_chapter_10
Insurance_type_ID	Secondary_Diagnoses_icd_10_chapter_17
Main_Diagnosis_second	Secondary_Diagnoses_icd_10_chapter_3
Occupation_ID	
Main_Diagnosis_first	
NUTS2_region_of_hospital	
Admission_criteria_ID	
**Target "is deceased"**	
had_intensive_care	Acute_case_or_emergency
Sex	is_romanian
Main_Procedure	Citizenship_ID
Admission_ward_ID	Secondary_Diagnoses_icd_10_chapter_12
Accident_ID	Secondary_Diagnoses_icd_10_chapter_4
Admission_type_ID	Secondary_Diagnoses_icd_10_chapter_19
Ventilation_hours	Secondary_Diagnoses_icd_10_chapter_11
Age	Secondary_Diagnoses_icd_10_chapter_20
Governmental_measures_randomized_phase_ID	Secondary_Diagnoses_icd_10_chapter_16
NUTS2_region_of_patient	Secondary_Diagnoses_icd_10_chapter_2
Date_of_main_procedure	Secondary_Diagnoses_icd_10_chapter_0
Date_of_main_procedure_day_of_week	Secondary_Diagnoses_icd_10_chapter_8
Date_of_main_procedure_hour_of_day	Secondary_Diagnoses_icd_10_chapter_7
Discharge_ward_ID	Secondary_Diagnoses_icd_10_chapter_9
has_COVID-19	Secondary_Diagnoses_icd_10_chapter_13
Secondary_Procedure	Secondary_Diagnoses_icd_10_chapter_22
has_COVID-19_suspected_or_performed_test	Secondary_Diagnoses_icd_10_chapter_18
Medical_Specialist_ID	Secondary_Diagnoses_icd_10_chapter_5
Days_spent_in_the_intensive_care_unit	Secondary_Diagnoses_icd_10_chapter_1
had_ventilation	Secondary_Diagnoses_icd_10_chapter_6
County_administrative_region_of_patient	Secondary_Diagnoses_icd_10_chapter_21
Suspected_Diagnosis	Secondary_Diagnoses_icd_10_chapter_15

Education_level_ID	Secondary_Diagnoses_icd_10_chapter_14
has_positive_test	Secondary_Diagnoses_icd_10_chapter_10
Hospital_ID	Secondary_Diagnoses_icd_10_chapter_17
Insurance_type_ID	Secondary_Diagnoses_icd_10_chapter_3
Main_Diagnosis_second	
Occupation_ID	
NUTS2_region_of_hospital	
Admission_criteria_ID	
**Target "acute case or emergency"**	
Sex	Secondary_Diagnoses_icd_10_chapter_20
Accident_ID	Secondary_Diagnoses_icd_10_chapter_16
Admission_type_ID	Secondary_Diagnoses_icd_10_chapter_2
Age	Secondary_Diagnoses_icd_10_chapter_0
Governmental_measures_randomized_phase_ID	Secondary_Diagnoses_icd_10_chapter_8
NUTS2_region_of_patient	Secondary_Diagnoses_icd_10_chapter_7
Secondary_Procedure	Secondary_Diagnoses_icd_10_chapter_9
has_COVID-19_suspected_or_performed_test	Secondary_Diagnoses_icd_10_chapter_13
County_administrative_region_of_patient	Secondary_Diagnoses_icd_10_chapter_22
Education_level_ID	Secondary_Diagnoses_icd_10_chapter_18
has_positive_test	Secondary_Diagnoses_icd_10_chapter_5
Insurance_type_ID	Secondary_Diagnoses_icd_10_chapter_1
NUTS2_region_of_hospital	Secondary_Diagnoses_icd_10_chapter_6
is_romanian	Secondary_Diagnoses_icd_10_chapter_21
Citizenship_ID	Secondary_Diagnoses_icd_10_chapter_15
Secondary_Diagnoses_icd_10_chapter_12	Secondary_Diagnoses_icd_10_chapter_14
Secondary_Diagnoses_icd_10_chapter_4	Secondary_Diagnoses_icd_10_chapter_10
Secondary_Diagnoses_icd_10_chapter_19	Secondary_Diagnoses_icd_10_chapter_17
Secondary_Diagnoses_icd_10_chapter_11	Secondary_Diagnoses_icd_10_chapter_3
**Target "has COVID-19"**	
Main_Procedure	Secondary_Diagnoses_icd_10_chapter_4
Admission_ward_ID	Secondary_Diagnoses_icd_10_chapter_19

Admission_Date	Secondary_Diagnoses_icd_10_chapter_11
Admission_Date_day_of_week	Secondary_Diagnoses_icd_10_chapter_20
Admission_Date_hour of day	Secondary_Diagnoses_icd_10_chapter_16
Admission_type_ID	Secondary_Diagnoses_icd_10_chapter_2
Age	Secondary_Diagnoses_icd_10_chapter_0
Governmental_measures_randomized_phase_ID	Secondary_Diagnoses_icd_10_chapter_8
NUTS2_region_of_patient	Secondary_Diagnoses_icd_10_chapter_7
Date_of_main_procedure	Secondary_Diagnoses_icd_10_chapter_9
Date_of_main_procedure_day_of_week	Secondary_Diagnoses_icd_10_chapter_13
Date_of_main_procedure_hour_of_day	Secondary_Diagnoses_icd_10_chapter_3
State_at_discharge_ID	Secondary_Diagnoses_icd_10_chapter_18
Medical_Specialist_ID	Secondary_Diagnoses_icd_10_chapter_5
is_cured_or_ameliorated	Secondary_Diagnoses_icd_10_chapter_1
County_administrative_region_of_patient	Secondary_Diagnoses_icd_10_chapter_6
Education_level_ID	Secondary_Diagnoses_icd_10_chapter_21
Hospital_ID	Secondary_Diagnoses_icd_10_chapter_15
Main_Diagnosis_second	Secondary_Diagnoses_icd_10_chapter_14
Occupation_ID	Secondary_Diagnoses_icd_10_chapter_10
NUTS2_region_of_hospital	Secondary_Diagnoses_icd_10_chapter_17
Admission_criteria_ID	
Acute_case_or_emergency	
Secondary_Diagnoses_icd_10_chapter_12	
