# Supplementary figures and images for: AutoML-Driven Insights into Patient Outcomes and Emergency Care During Romania’s First Wave of COVID-19
Source: Bioengineering (Basel). 2024 Dec 15;11(12):1272. doi: 10.3390/bioengineering11121272 (PMC11673140; doi:10.3390/bioengineering11121272)

(a)

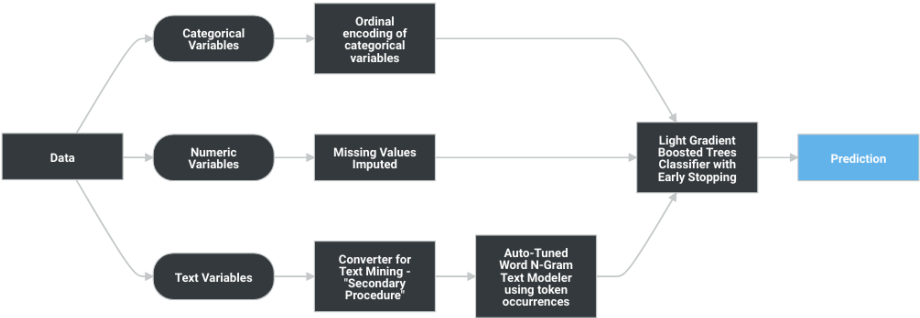

(b)

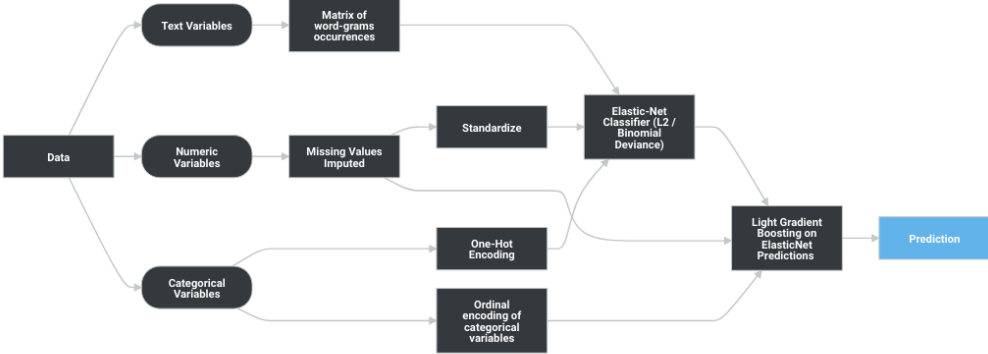

(c)

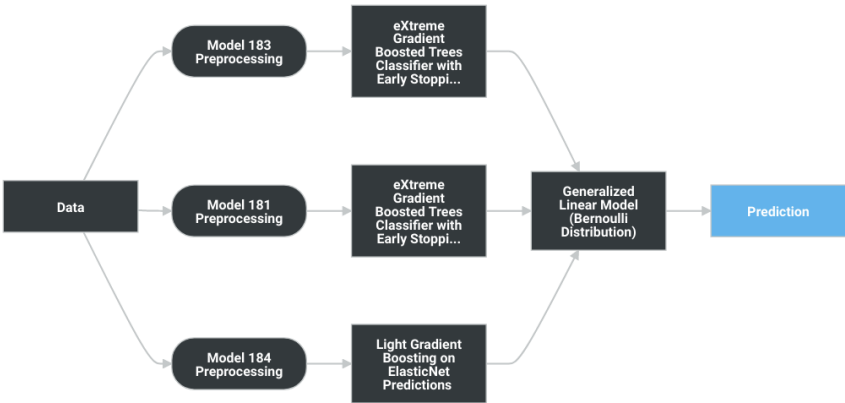

(d)

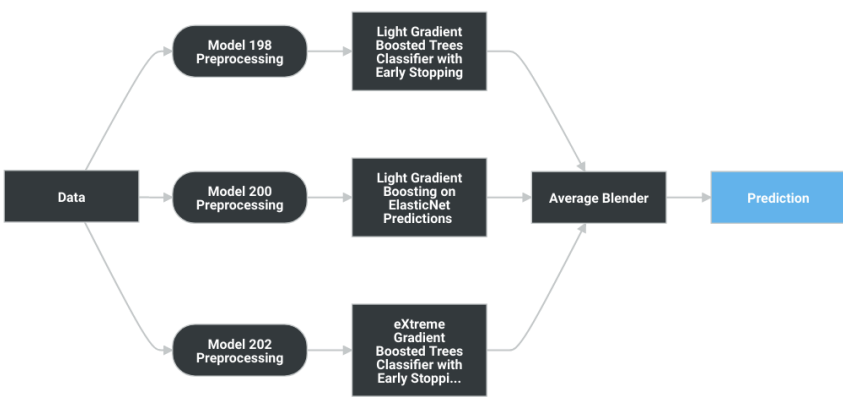

Supplement: Supplementary file 1 [file bioengineering-11-01272-s001.zip › Figure S1 - Bioengineering - AutoML COVID.pdf]

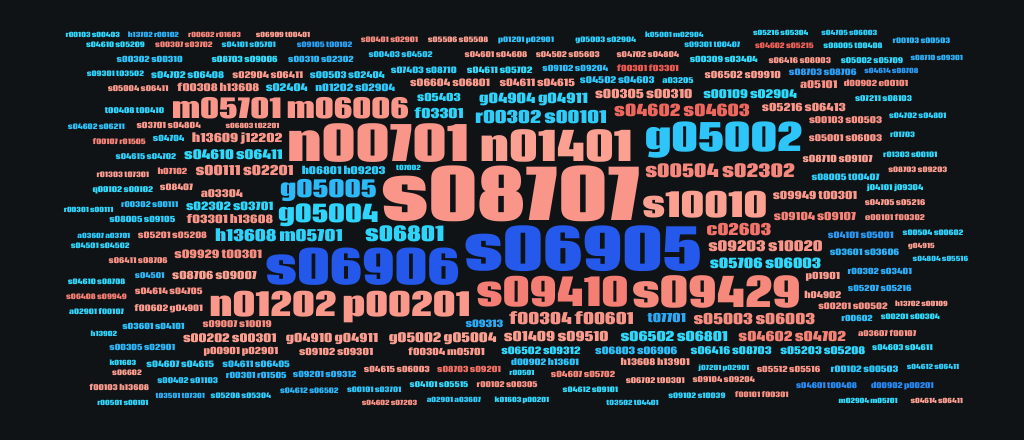

Supplement: Supplementary file 1 [file bioengineering-11-01272-s001.zip › Figure S2 - Bioengineering - AutoML COVID.png]

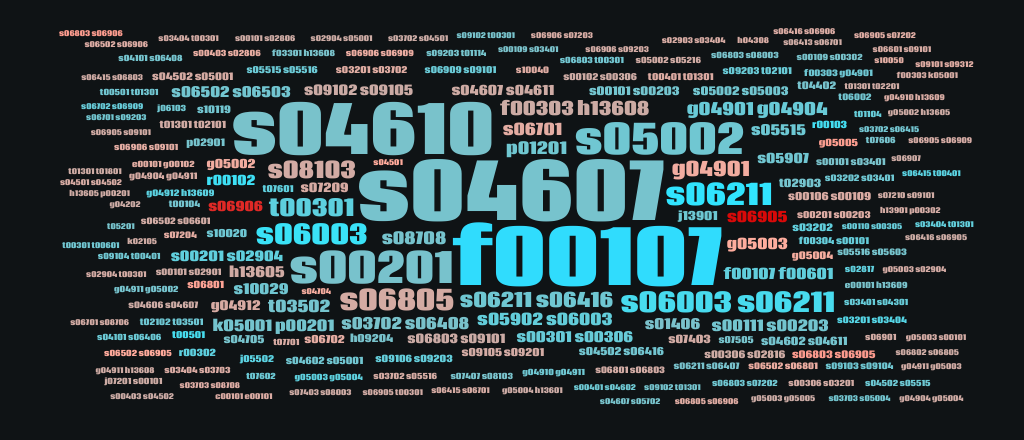

Supplement: Supplementary file 1 [file bioengineering-11-01272-s001.zip › Figure S3 - Bioengineering - AutoML COVID.png]

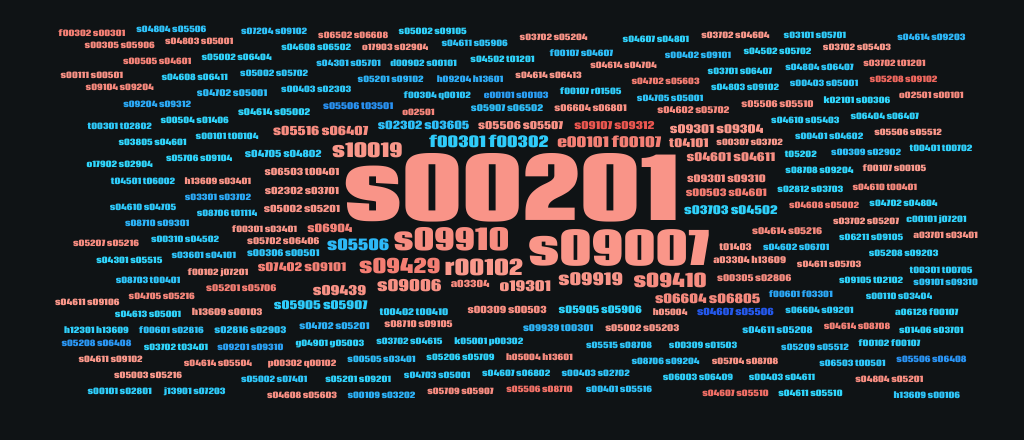

Supplement: Supplementary file 1 [file bioengineering-11-01272-s001.zip › Figure S4 - Bioengineering - AutoML COVID.png]
